# Reversal of bleomycin-induced rat pulmonary fibrosis by a xenograft of human umbilical mesenchymal stem cells from Wharton's jelly

**DOI:** 10.7150/thno.33741

**Published:** 2019-09-17

**Authors:** Kuo-An Chu, Shih-Yao Wang, Chang-Ching Yeh, Tz-Win Fu, Yu-Yi Fu, Tsui-Ling Ko, Mei-Miao Chiu, Tien-Hua Chen, Pei-Jiun Tsai, Yu-Show Fu

**Affiliations:** 1 Division of Chest Medicine, Department of Internal Medicine, Kaohsiung Veterans General Hospital, Kaohsiung, Taiwan, ROC; 2 Department of Nursing, Shu-Zen Junior College of Medicine and Management, Kaohsiung, Taiwan, ROC; 3 Institute of Anatomy and Cell Biology, School of Medicine, National Yang-Ming University, Taipei, Taiwan, ROC; 4 Department of Obstetrics and Gynecology, Taipei Veterans General Hospital, Taipei, Taiwan, ROC; 5 Department of Obstetrics and Gynecology, National Yang-Ming University, Taipei, Taiwan, ROC; 6 Department of Nurse-Midwifery and Women Health, National Taipei University of Nursing and Health Sciences, Taipei, Taiwan, ROC; 7 Department of Laboratory Medicine, Taipei Veterans General Hospital, Taipei, Taiwan, ROC; 8 Department of Business Administration, Nan-Kai University of Technology, Nantou, Taiwan, ROC; 9 School of Medicine, I-Shou University, Kaohsiung, Taiwan, ROC; 10 Department of Medicine, Mackay Medical College, New Taipei, Taiwan, ROC; 11 Trauma Center, Department of Surgery, Veterans General Hospital, Taipei, Taiwan, ROC; 12 Division of General Surgery, Department of Surgery, Veterans General Hospital, Taipei, Taiwan, ROC.; 13 Department of Critical Care Medicine, Veterans General Hospital, Taipei, Taiwan, ROC; 14 Department of Anatomy and Cell Biology, School of Medicine, National Yang-Ming University, Taipei, Taiwan, ROC

**Keywords:** Pulmonary fibrosis, Umbilical mesenchymal stem cells, Transplantation

## Abstract

Pulmonary fibrosis (PF) is a progressive and irreversible condition with various causes, and no effective treatment has been found to rescue fibrotic lungs. Successful recovery from PF requires inhibiting inflammation, promoting collagen degradation and stimulating alveolar regeneration. Human umbilical mesenchymal stem cells (HUMSCs) not only regulate immune responses but also synthesize and release hyaluronan to improve lung regeneration. This study investigated the feasibility of HUMSC engraftment into rats with bleomycin (BLM)-induced PF to explore HUMSC therapeutic effects/outcomes.

**Methods:** A unique BLM-induced left-lung-dominated PF animal model was established. Rats were transplanted with low-dose (5×10^6^) or high-dose (2.5×10^7^) HUMSCs on Day 21 after BLM injection. Combinations in co-culture of pulmonary macrophages, fibroblasts, HUMSCs treated with BLM and the same conditions on alveolar epithelia versus HUMSCs were evaluated.

**Results:** Rats with high-dose HUMSC engraftment displayed significant recovery, including improved blood oxygen saturation levels and respiratory rates. High-dose HUMSC transplantation reversed alveolar injury, reduced cell infiltration and ameliorated collagen deposition. One month posttransplantation, HUMSCs in the rats' lungs remained viable and secreted cytokines without differentiating into alveolar or vascular epithelial cells. Moreover, HUMSCs decreased epithelial-mesenchymal transition in pulmonary inflammation, enhanced macrophage matrix-metallopeptidase-9 (MMP-9) expression for collagen degradation, and promoted toll-like receptor-4 (TLR-4) expression in the lung for alveolar regeneration. In coculture studies, HUMSCs elevated the MMP-9 level in pulmonary macrophages, released hyaluronan into the medium and stimulated the TLR-4 quantity in the alveolar epithelium.

**Principal Conclusions:** Transplanted HUMSCs exhibit long-term viability in rat lungs and can effectively reverse rat PF.

## Introduction

Heterogeneous factors cause lung tissue damage**,** such as smoking, aging, air pollution, bacteria, viruses, free radicals, radiation, and chemotherapeutic agents, as well as by epigenetic/hereditary factors 1-4. The number of functional alveoli decreases and the alveolar compartments are gradually replaced by fibrotic tissues during pathological progression, causing pulmonary fibrosis (PF) 5. This irreversible consequence causes a progressive deterioration in lung functionality 5, 6. Critically, the mortality rate for PF is increasing every year 4. To date, no effective therapy has been developed for PF.

Type II alveolar epithelial cells (AEC2s) proliferate and transdifferentiate into Type I cells (AEC1s) at a slow pace to repair damaged alveoli 7, 8. However, if this process becomes dysregulated, it can lead to the release of numerous inflammatory mediators and infiltration of immune cells and can trigger the epithelial-mesenchymal transition of AEC2s; transformed AEC2s may then differentiate into active myofibroblasts. These myofibroblasts not only express α-smooth muscle actin (α-SMA) but also produce components of the extracellular matrix (ECM), leading to fibrosis in lung tissue. The excessive deposition of ECM proteins is a hallmark of PF 9-13.

Various animal models for PF have been developed 14-23; intratracheal bleomycin (BLM) injection is a frequent application for PF induction because bleomycin-induced PF is patchy and sporadic, appearing in an unpredictable pattern similar to that in humans clinically 18-21. A meta‑analysis of six studies involving 228 model rats proposed that higher‑quality and more rigorous studies are required to estimate the potential utility of stem cells for the treatment of BLM‑induced PF 16. In order to evaluate precisely the therapeutic effect of transplanted stem cells on chronic fibrosis stage or PF, and simultaneously keep the experimental animals survived, we modified the BLM-induced PF model to develop such a severe, stable, and one-sided (left-lobe) PF with consistent reproducibility in this study.

Scientists have invested great effort into investigating the use of stem cells for PF therapy, including adipose tissue mesenchymal stem cells (MSCs) 24, bone marrow MSCs 16, lung spheroid cells 25-27, and human umbilical MSCs (HUMSCs) from Wharton's jelly 20. Although the origins of stem cells in the aforementioned studies varied, the results indicated that transplantation of stem cells can alleviate inflammation and thereby mitigate or prevent PF. However, in most relevant studies, stem cells were engrafted either immediately, one day, or acute phase after the induced lung damage, thus focusing on the capability of the stem cell in rescuing acute pneumonia or stopping PF from developing 28. In contrast, in clinical practice, most patients attending clinics have already developed respiratory problems manifesting various degrees of PF. Therefore, reversing the functionality and status of PF is a therapeutic imperative.

We have demonstrated that HUMSCs can survive in different organs of rats, suggesting that HUMSCs do not induce xenograft rejection and can serve as an excellent stem cell source 29-38. In addition, HUMSCs are deemed superior for the treatment of PF, as the highest amount of hyaluronan (HA) is found in the umbilical cord 39, 40. HA existence and toll-like receptor-4 (TLR-4) expression on AEC2s play important roles in lung renewal as well as inflammatory regulators for immune cells 41-44.

In this study, we investigated the therapeutic effect of HUMSC transplantation on BLM-induced PF in rats. BLM was injected intratracheally to induce a unique left-lobe-dominated PF model. Subsequently, low (5×10^6^) and high (2.5×10^7^) doses of HUMSCs were transplanted intratracheally on Day 21 after BLM injection. Our objective was to determine whether the transplantation of HUMSCs reverses PF.

## Methods

The use of human umbilical cords and laboratory animals in this study was approved by the Research Ethics Committee of Taipei Veterans General Hospital and the Animal Research Committee of National Yang-Ming University.

### Isolation and culture of human umbilical mesenchymal stem cells (HUMSCs)

Human umbilical cords were collected aseptically and kept at 4 °C in Hank's Balanced Salt Solution (HBSS). The mesenchymal tissue (Wharton's jelly) was cut into small pieces and centrifuged at 4000 rpm for 5 min. The umbilical mesenchymal tissue was treated with collagenase and trypsin, followed by the addition of fetal bovine serum (FBS; Gibco 10437-028) to stop the reaction; at that point, the umbilical mesenchymal cells were fully processed into HUMSCs. Finally, HUMSCs were then used directly for cultures in 10% FBS Dulbecco's modified Eagle's medium (DMEM) or stored in liquid nitrogen for later use. The double time of HUMSCs was around 18- 24 h. HUMSCs were collected between the tenth and fifteenth passages for transplantation into rats in this study. In a previous study, similarly processed HUMSCs were found to express high levels of matrix receptors (CD44, CD105), integrin (CD29, CD51) and mesenchymal stem cell markers (SH2, SH3) but did not express hematopoietic lineage markers (CD34, CD45) 45 (Supplemental Figure 1B). HUMSCs did not display any chromosomal abnormality in the karyotype of HUMSCs *in vitro* using CytoScan 750K Array (Affymetrix) (Supplemental Figure 1A).

### Establishing an animal model for PF in the left lung

A serial experiment was performed to determine the load of intratracheal BLM required to produce a severe, stable, and one-sided (left-lobe) PF with consistent reproducibility (Supplemental Figure 1D). Following confirmation of anesthesia depth, male Sprague Dawley (SD) rats received 2 Unit/2 mg BLM/250 g body weight (Nippon Kayaku Co., Ltd.) in 200 μL phosphate buffered saline (PBS) by intratracheal injection and were then rotated to the left side by 60° for 90 min.

### HUMSC transplantation

HUMSCs were treated with 0.05% trypsin-EDTA (Gibco 15400-054) for 2.5 min. Cells were then collected and washed twice with 10% FBS DMEM. The pelleted cells were subsequently suspended at a concentration of 5 × 10^6^ or 2.5 × 10^7^ in 200 μL of 0.01 M PBS. On Day 21 after intratracheal BLM, rats were treated with 5×10^6^ or 2.5×10^7^ HUMSCs by intratracheal transplantation.

### Animal groups

The animals were randomized to the following treatment:

Normal group (n=17) rats were intratracheally injected with 200 μL of PBS instead of BLM. PBS was intratracheally administered to the rats again on Day 21.

BLM group (n=25) rats received an intratracheal injection with 2 mg of BLM and were sacrificed on Days 7, 14, 21, 28 and 49. On Day 21 after BLM injection, PBS was intratracheally administered to the rats.

BLM+HUMSCs (LD) group (n=12) rats received 2 mg of BLM and then intratracheal transplantation of 5×10^6^ (low-dose) HUMSCs on Day 21 after BLM injection.

BLM+HUMSCs (HD) group (n=20) rats received 2 mg of BLM and then intratracheal transplantation of 2.5×10^7^ (high-dose) HUMSCs on Day 21 after BLM injection.

**Figure 1:**
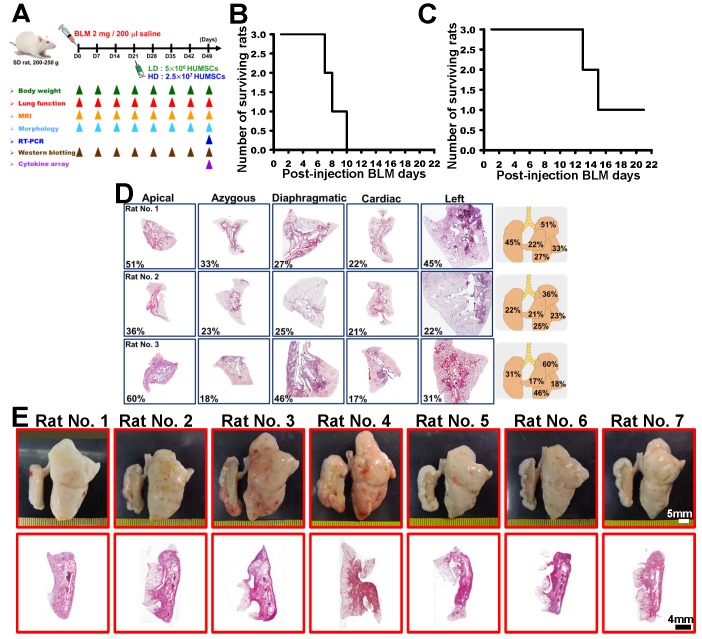
** A specific one-sided left lung-dominated PF animal model was successfully established in rats.** Experimental flowchart for inducing PF in rats' left lungs, the transplantation of HUMSCs, and the time course for various experiments in this study (A). BLM-induced PF in SD rats. Short Kaplan-Meier survival curves of 5 or 3 mg BLM injection indicated dose toxicity (B and C). A 2 mg BLM general intratracheal injection (n=3) showed inconsistent degrees of PF in all lobes after 49 days (D, H&E stains, right graphs % summary). There was no distinct change in appearance, and the PF was less than 50% (D). A one-sided left lung PF animal model was designed to create a stable, reproducible, consistent disease animal model. The results from the 2 mg/rat test group (n=7) in overall lung appearance and H&E staining demonstrated that a one-sided left lung PF animal model was successfully established in rats (E).

The experimental flowchart is displayed in Figure 1A.

### Sacrifice and perfusion fixation of experimental animals

Animals were anesthetized and then perfused with 0.01 M PBS. Both lungs were removed and immersed in a fixation solution with 4% paraformaldehyde (Sigma 10060) and 7.5% picric acid (Sigma 925-40). The left and right lungs were postfixed in the fixative solution and then subjected to paraffin embedding. Lung tissue blocks were sectioned into 5 μm slices. A serial sagittal section was performed from the outermost lateral side. Ten slices were numbered consecutively and placed on slides for various immunohistochemistry (IHC) examinations (Supplemental Figure 2).

### Hematoxylin and eosin (H&E) staining

Lung tissue sections were immersed in hematoxylin solution (Muto Pure Chemicals Co., Ltd.; No. 3008-1) and eosin solution (Muto Pure Chemicals Co., Ltd.; No. 3200-2). The left lung volume, percentage of cell infiltration area and air space (Supplemental Figure 2, Column A), stained using H&E, was quantified by the average red signals in every left lung section (infiltration and air space). The total left-lobe volume was the summation of all H&E signals in the collected images. The quantification method for alveoli circumference was based on the inner space peripheral lengths of each empty alveoli in unit area (N=7, 30 images each), analyzed with Image-Pro software.

### Sirius red stain

Lung tissue sections were stained in 0.1% Sirius red (Sigma 2610-10-8) in picric acid and then photographed using optical microscopy. The percentage of collagen deposition (Supplemental Figure 2, Column B), stained by Sirius red, was quantified by the average number of red signals in every left lung section. Image signals (N=7, 30 images each) were analyzed with Image-Pro software.

### Magnetic resonance imaging (MRI)

Rat lung images were obtained through MRI (Bruker BioSpec 70/30) at the Instrumentation Center of National Taiwan University. The thoracic cavities of the rats were scanned from rostral to caudal and photographed horizontally every 1.5 mm until the whole thoracic cavity had been scanned. Because the first image obtained in each rat varied in position, the total number of images in the horizontal plane was 20 to 25.

Image-Pro Plus software was employed, and the carina of the trachea was used as a landmark for image positioning. Five images contained the slice from the level of the carina, and two slices before and after the carina were summed for quantification of the black alveolar space to represent the left lung alveolar volume of the rats (Supplemental Figures 3- 6).

### Pulmonary function testing in experimental animals

#### Determination of arterial blood oxygen saturation

After shaving the fur on the rats' front legs, they were anesthetized with isoflurane (Baxter 228-194) for 40 min. The shaved legs were clipped with a pulse oximeter (Rossmax SB100) for measuring the arterial blood oxygen saturation as modified by Rancourt's method 46.

#### Determination of pulmonary respiratory rates

Experimental animals were placed in a closed cylinder-like detection chamber (whole body plethysmograph, emka Technologies), in which the alterations in breath flow were recorded for 15 min using the BIOPAC BSL 4.0 MP45 software package. The respiratory rates of the rats were quantified while they were still.

### Tracing the viability and distribution of HUMSCs with bisbenzimide

To trace the viability and distribution of the implanted HUMSCs, cells were labeled with 1 μg/mL bisbenzimide (Sigma B2883) for 48 h before trypsinization with 0.05% Trypsin (Gibco 15400-054) and subsequent transplantation. Transplantation of HUMSCs was performed 21 days after BLM injection. After 1 month, the rats were sacrificed and perfused. Left and right lungs were obtained and immersed in fixative. Lungs were embedded in tissue-freezing medium and cryosectioned at 30 μm with a cryostat at -20 °C. After mounting with Fluoromount Aqueous Mounting Medium (Sigma F4680), the distribution of the HUMSCs was directly observed under a fluorescence microscope.

### Immunohistostaining to label the HUMSCs using anti-human nuclear antigen

IHC was applied to observe the HUMSCs. Samples were reacted with mouse anti-human specific nuclei antigen (Millipore MAB1281, 1:100) at 4 °C for 36- 42 h and reacted with secondary antibody at room temperature for 60 min. Finally, the color was developed with 3,3'diaminobenzidine (DAB) to trace the viability of the HUMSCs in the rat lungs.

### Reverse transcription polymerase chain reaction (RT-PCR)

The left lung tissues from the Normal, BLM-treated and BLM+HUMSCs (HD) groups on Day 49 were subjected to RT-PCR to identify the complementary DNA (cDNA) of human surfactant protein D (SP-D) and human platelet endothelial cell adhesion molecule (PECAM-1) to examine whether the HUMSCs had differentiated into human alveolar or vascular endothelial cells. Total RNA of the lung tissue was extracted using TRIzol^®^ Reagent (Invitrogen^®^ 15596-018). After quantification, 2 μg of RNA was subjected to reverse transcription. After cDNA was synthesized, 1 μL of cDNA was used for PCR with the following primers:

(1) Human SP-D:

F: 5'-AGGAGCAAAGGGAGAAAGTGG-3'

R: 5'-GCTGTGCCTCCGTAAATGGT-3' Product length: 197 bps

(2) Human PECAM-1:

F: 5'-TCAAGAAAAGCAACACAGTCC-3'

R: 5'-ACTCCGATGATAACCACTGC-3' Product length: 652 bps

(3) Rat *Acta*2

F: 5'-GCCATCAGGAACCTCGAGAA-3'

R: 5'- AGTTGGTGATGATGCCGTGT-3' Product length: 273 bps

(4) Rat MMP-9

F: 5' -TTCAAGGACGGTCGGTATT -3'

R: 5'-CTCTGAGCCTAGACCCAACTTA-3'

Product length: 228 bps

(5) Human MMP-9

F: 5'-GCCACTACTGTGCCTTTGAGTC-3'

R: 5'-CCCTCAGAGAATCGCCAGTACT-3'

Product length: 125 bps

(6) Rat TLR-4

F: 5'-ATCATCCAGGAAGGCTTCCA -3'

R: 5'- GCTGCCTCAGCAAGGACTTCT-3' Product length: 181 bps

(7) Human TLR-4

F: 5'-CCCTGAGGCATTTAGGCAGCTA-3'

R: 5'-AGGTAGAGAGGTGGCTTAGGCT-3'

Product length: 126 bps

(8) Human glyceraldehyde-3-phosphate dehydrogenase (GAPDH)

F: 5'-TTCCACCCATGGCAAATTCCATGG-3'

R: 5'-GGTCAGGTCCACCACTGACACG-3' Product length: 589 bps

(9) Rat GAPDH

F: 5'-CTCTACCCACGGCAAGTTCAAC-3'

R: 5'-GGTGAAGACGCCAGTAGACTCCA-3

Product length: 160 bps

The temperature settings were as follows: enzyme activation for 10 min at 95 °C; denaturing for 30 s at 95 °C; annealing for 30 s at 56- 60 °C; and extension for 1 min at 72 °C. After 35 cycles of PCR, samples were finished for 5 min at 72 °C. The PCR products were analyzed using 2% agarose gel electrophoresis and visualized in an ultraviolet transilluminator.

### Quantitative real-time PCR

Extraction of total RNA from lung were performed with RNAiso Plus reagent and further reverse-transcribed using a PrimeScript RT reagent kit (BIONOVAS HiScript I First Strand cDNA Synthesis Kit, AM0675-0050). SYBR-Green mix (Luna Universal qPCR Master Mix, M3003) was used to carry out quantitative PCR according to the manufacturer's instructions. Target gene expression was normalized to β-actin levels in respective samples as an internal control and calculated using the 2-ΔΔCq method, and the relative mRNA expression was further calculated through normalizing to the Normal group.

### Immunohistostaining

Lung slices (with recovered antigens) were reacted with primary antibodies (mouse anti-rat ED1 antibody for total macrophage [Millipore MAB1435]; rabbit anti- rat iNOS antibody for M1 macrophage [Abcam ab15323]; mouse anti- rat CD163 antibody for M2 macrophage [BioRad MCA342R]; rabbit anti- human Podoplanin gp36 antibody for human alveolar type I cell [Abcam 236529]; mouse anti-toll-like receptor-4 antibody [TLR-4, Abcam ab30667, specific for rat and human]; mouse anti-α-smooth muscle actin antibody for myofibroblast [SMA, Sigma A2547]) at 4°C for 12- 18 h and then reacted with secondary antibodies. Samples were then reacted with avidin-biotinylated-horseradish peroxidase complex (ABC Kit, Vector Laboratories) and finally developed with DAB. Finally, the numbers of M1 macrophage or M2 macrophage were counted from ten optic fields in three left lung sections of each group.

### Western blotting

Lung tissue samples from each group were loaded separately into individual wells. The polyvinylidene fluoride (PVDF) paper was reacted with primary antibodies (mouse anti-α-SMA antibody, rabbit anti-matrix metallopeptidase 2 antibody [MMP-2, Abcam ab92536, specific for rat and human], rabbit anti- MMP-9 antibody [Abcam ab76003, specific for rat and human], mouse anti-toll-like receptor-4 antibody [TLR-4, Abcam ab30667, specific for rat and human], and mouse anti-β-actin antibody [Sigma A5411] for internal control) at 4 °C for 12- 18 h. Subsequently, the membrane was reacted with the corresponding secondary antibodies at room temperature for 1 h. The protein bands were quantified using Image J software and normalized using individual internal controls for comparison.

### Total MMPs activity assay

Tatal MMPs activity was assayed using Gelatinase Assay Kit (Fluorometric) (Bio Vision, Catalog # K444-100). Lung tissue lysates were collected Enzyme activity was determined using a fluorescently labeled peptide as substrate according to the manufacturer's instructions. Briefly, 50 µl MMPs substrate solution (100 µM) was mixed with 50 µl lung tissue lysates in a black 96 well microtiter plate. The change in fluorescence (expressed as relative fluorescence units) was measured at Ex/Em = 490 nm/520 nm every 5 min for up to 1 h using a luminescence spectrometer.

### Zymography

Gelatinolytic activity of MMP-9 and MMP-2 in the left lung of rats on Day 49 were assessed by zymography. 25 µg of protein were mixed with non-reducing loading-buffer (0.5 M Tris pH 6.8, 20% SDS, 50 mM NaEDTA, 0.2% bromphenolblue, 10% glycerol) and loaded onto an 8% SDS-polyacrylamide gel (see SDS-PAGE) additionally containing 0.1% gelatine. Electrophoresis was performed as described. Gelatinolytic bands were developed through incubation in the reaction buffer (50 mM Tris pH 7.4, 5 mM CaCl_2_, 1 µM ZnCl_2_) for 16 h and subsequent staining with staining buffer (0.5% Coomassie G250, 30% EtOH, 10% acetic acid) and destaining buffer. Sizes of the clear bands were estimated by comparison with molecular weight marker on the same gel.

### Measurement of lung hydroxyproline

The collagen content in lungs of rat was measured using the hydroxyproline assay kit (BioVision, K226-100) with modifications from Reddy's method 47. In brief, the lungs were hydrolyzed with 10 N NaOH at 120 °C for 1 h. After neutralization with hydrochloric acid, the hydrolysates were diluted with distilled water. Hydroxyproline in the hydrolysates was assessed calorimetrically at 550 nm with *p*-dimethylaminobenzaldehyde.

### Bronchoalveolar lavage

Rats were anesthetized and their airways were lavaged two times with 2 ml saline/each, and 1 ml bronchoalveolar lavage fluid (BALF) was recovered. Total cell counts were determined using a hemocytometer.

### Human Cytokine Array and Rat Cytokine Array

To elucidate which human cytokines released from HUMSCs were involved in the treatment of rat PF, a human protein cytokine kit (AAH-CYT-2000, RayBio Human Cytokine Antibody Array C Series 2000, RayBiotech) was used to screen the expression of 174 human cytokines (n = 3/group). In addition, a RayBio® Rat Cytokine Antibody Array C2 (RayBio® AAR-CYT-2-8) was used to detect cytokines produced or secreted by the rat lung tissues (n = 3/group). The rats were deeply anesthetized and decapitated at 1 month after HUMSC transplantation. Lung were homogenized in lysis buffer and centrifuged at 1500 g to separate cell debris. The supernatant was harvested and then incubated for 2 h at room temperature with membranes containing an array of human cytokine antibodies. The levels of cytokine expression were determined by the intensities of immunoreactivity as relative to that of the standard controls using enhanced chemiluminescence according to the manufacturer's instructions.

### Alveolar cells and HUMSCs coculture system

To investigate whether HUMSCs promote the turnover of alveolar cells via TLR-4, the quantification of TLR-4 expression in alveolar cells was assayed using alveolar cells and a HUMSC coculture system. Western blotting was first conducted with an anti-proSPC antibody to confirm the existence of type II alveolar epithelial cells in L2 cells 48 (purchased from the Food Industry Research and Development Institute, Hsinchu; 60276, ATCC# CCL-149). First, we used the MTT assay to detect the ratio of cell survival in various cells after the administration of 0- 20 mg/mL BLM (Supplemental Figure 3B). L2 cells were cultured in 6-well plates (10^5^ cells/well) and treated with 100 μM TLR-4 inhibitor (C34, TOCRIS, 40592-88-9) 49 for 12 h, followed by replacement with a medium containing 1 mg/mL BLM and 100 μM TLR-4 inhibitor for 24 h. Subsequently, the medium containing only 100 μM TLR-4 inhibitor was used for coculture of alveolar cells and HUMSCs (10^5^) for 24 h. HUMSCs were cultured in transwells. Alveolar cells in each group were photographed to observe the cell number, and then the expression level of TLR-4 was quantified by western blotting.

### Pulmonary macrophages, fibroblasts and HUMSCs coculture system

A sequential coculture method was applied in this *ex vivo* experiment: the first coculture of pulmonary macrophages (upper transwell) and fibroblasts with/without PF induction (BLM) was performed. Then, macrophages alone (upper transwells) were transferred to a second coculture with HUMSCs (bottom) in a transwell plate. Pulmonary macrophages (NR8383, purchased from the Food Industry Research and Development Institute, Hsinchu; 60423, ATCC #CRL-2192) were cultured in 6-well plates (5×10^5^ cells/well), and fibroblasts (5×10^4^ cells) were cultured in transwells for coculture. Pulmonary macrophages and fibroblasts were treated with 0.5 mg/mL BLM for 24 h. Subsequently, the upper transwells containing macrophages were transferred to HUMSCs. With fresh DMEM, coculture of HUMSCs and pulmonary macrophages was incubated for 48 h. Finally, the quantity of MMP-9 in cell lysates of pulmonary macrophages was examined by western blotting.

### Statistical analysis

All data are presented as the mean ± standard error of the mean (SEM). One-way analysis of variance (ANOVA) was used to compare the means, and Fisher's least significant difference test was applied for multiple comparisons. A value of p < 0.05 was considered statistically significant.

## Results

### Animal model establishment of one-sided left lung PF in rats

To establish a one-sided lung PF model that could provide a severe, stable and one-sided (left-lobe) PF condition with consistent reproducibility, pilot studies in rats had tested the following: (1) dosage ranges of PF induction/rat survival, (2) uni-/bilateral lung lobe PF development, (3) induction methodology (general intratracheal injection *vs* side-specific intratracheal injection), (4) disease development (PF) time frame, and (5) PF severity in regimen rats. BLM in 5, 3 and 2 mg/rat doses were applied to rats by using general intratracheal injection into both lung lobes. In the 5 mg/rat and 3 mg/rat BLM dose groups, the toxicity of BLM overwhelmed the survival of the tested rats. All rats in the 5 mg/rat group were deceased between 7 and 10 days after injection and after 12- 15 days in the 3 mg/rat group (Figures 1B and 1C).

The 2 mg/rat test group survived more than 50 days; however, rats given 2 mg using general intratracheal injection showed a PF discrepancy in all lung lobes; e.g., the damaged area (H&E stained) in the left lobes displayed 45%, 22% and 31% cell infiltration, in the three rats studied (Figure 1D). To avoid such discrepancies in PF induction in the animal model, a side-specific (left-lobe) induction method was designed to create a stable, reproducible, consistent disease animal model. The results from the 2 mg/rat tested group (sacrificed at Day 49) in overall lung appearance and H&E staining demonstrated that a one-sided left lung PF animal model met the disease model requirements for the experiment (Figure 1E, n=7).

### HUMSC transplantation reduces collagen deposits in the PF lung

**Figure 2:**
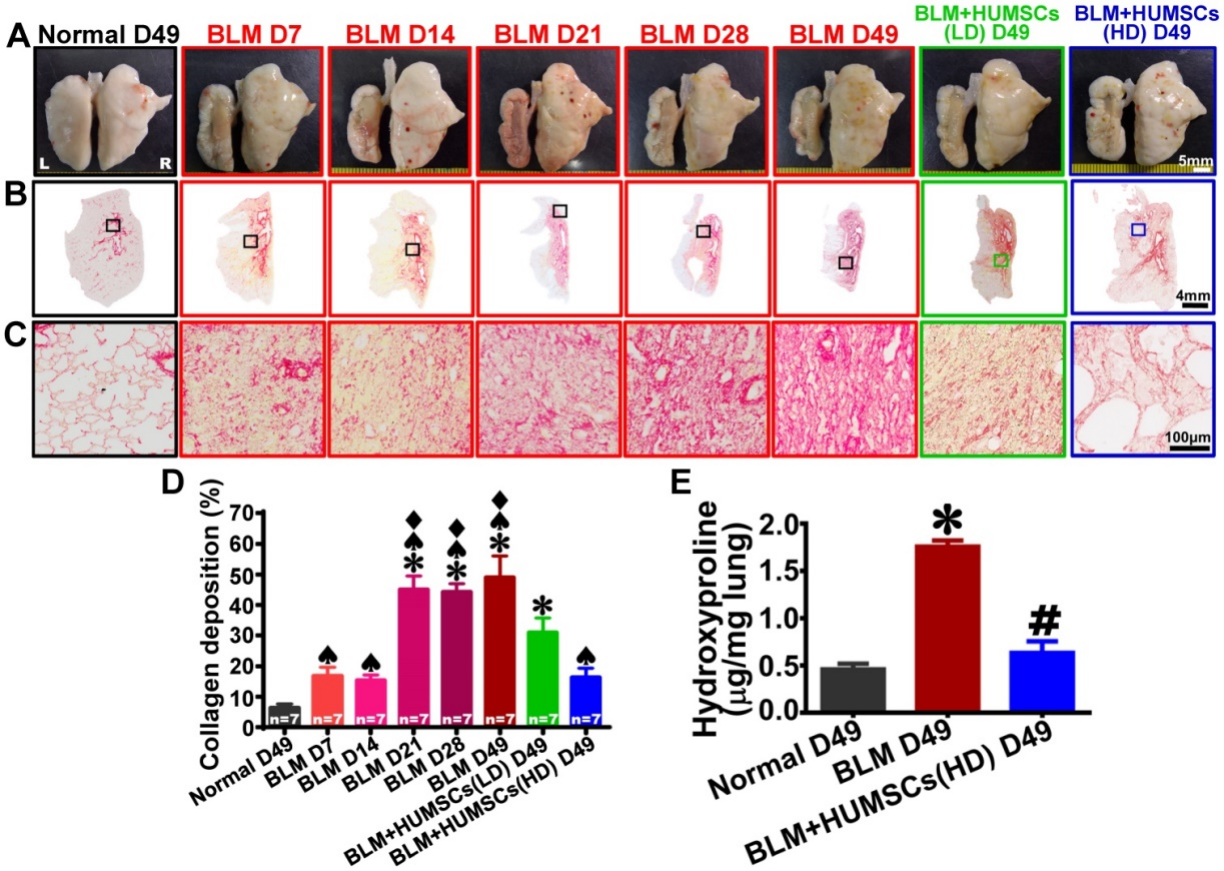
** HUMSC transplantation reduced collagen accumulation in PF rats.** (A) The overall appearances of the anterior views of the left and right lungs were obtained. The white alveolar structures were intact and smooth in both the left and right lungs of the Normal group. From Day 7 to 49 after BLM injection, the left lung markedly shrank, and healthy alveoli were only present at the perimeters of the left lungs. Transplantation of high doses of HUMSCs significantly alleviated the shrinking of the left lungs in rats with PF. (B and C) Left lung sections were stained with Sirius red to indicate the presence of collagen. From low to high magnification, large red regions appeared in the left lungs after Day 21 and were maintained until Day 49. The transplantation of HUMSCs reduced collagen in the left lung (D), n=7 animals per group. (E) Hydroxyproline content significantly increased in the rats of BLM group, whereas hydroxyproline level substantially decreased in the rats of BLM+HUMSCs group. ✱* vs* the Normal group, p < 0.05.** #** *vs* the BLM group, p < 0.05. ♠ *vs* the BLM+HUMSCs (LD) group, p < 0.05. ⧫ *vs* the BLM+HUMSCs (HD) group, p < 0.05.

Intact, smooth and white alveolar structures were observed in the left and right lungs of the Normal group. On Day 7 after BLM injection, no alveoli were present in the central region of the left lung, but pathological tissues and scar tissues were present. Healthy alveoli were only present at the margins of the left lung. On Days 14, 21 and 28 after BLM injection, the scar tissue in the central region of the left lungs had shriveled, and this condition remained until Day 49 (Figure 2A). The appearance of the lungs in the BLM+HUMSCs(LD) group showed no significant improvement, whereas there was substantial enhancement in the BLM+HUMSCs (HD) (Figure 2A).

The left lung sections were stained with Sirius red to label collagen red (Figures 2B and 2C). Collagen appeared only near the bronchus and blood vessels in the Normal group. Collagen exhibited slight increases on Days 7 and 14 after BLM injection, a considerable enhancement on Day 21, and a stable plateau until Day 49 (Figure 2D). Therefore, HUMSCs were transplanted into the tracheas on Day 21 after BLM injection.

The percentage of collagen deposition in the left lungs in the BLM+HUMSCs (LD) group significantly decreased compared with that in the BLM group on Day 49, whereas it remained significantly higher than that in the Normal group on Day 49 (Figure 2D). Collagen deposition in the BLM+HUMSCs (HD) group was not only lower than that in the BLM group (Day 49) but also not significantly different from that of the Normal group (Day 49) (Figure 2D).

Hydroxyproline assay was applied to calculate the collagen deposition in lung on Day 49. There was a significant up-regulation in collagen deposition in the BLM group compared with that of Normal group. HUMSCs transplantation resulted in a significant reduction of collagen deposition when compared with BLM group (Figure 2E).

### HUMSC transplantation ameliorates left lung shrinkage and restores alveolar structures to improve alveolar gas exchange in rats with PF

**Figure 3:**
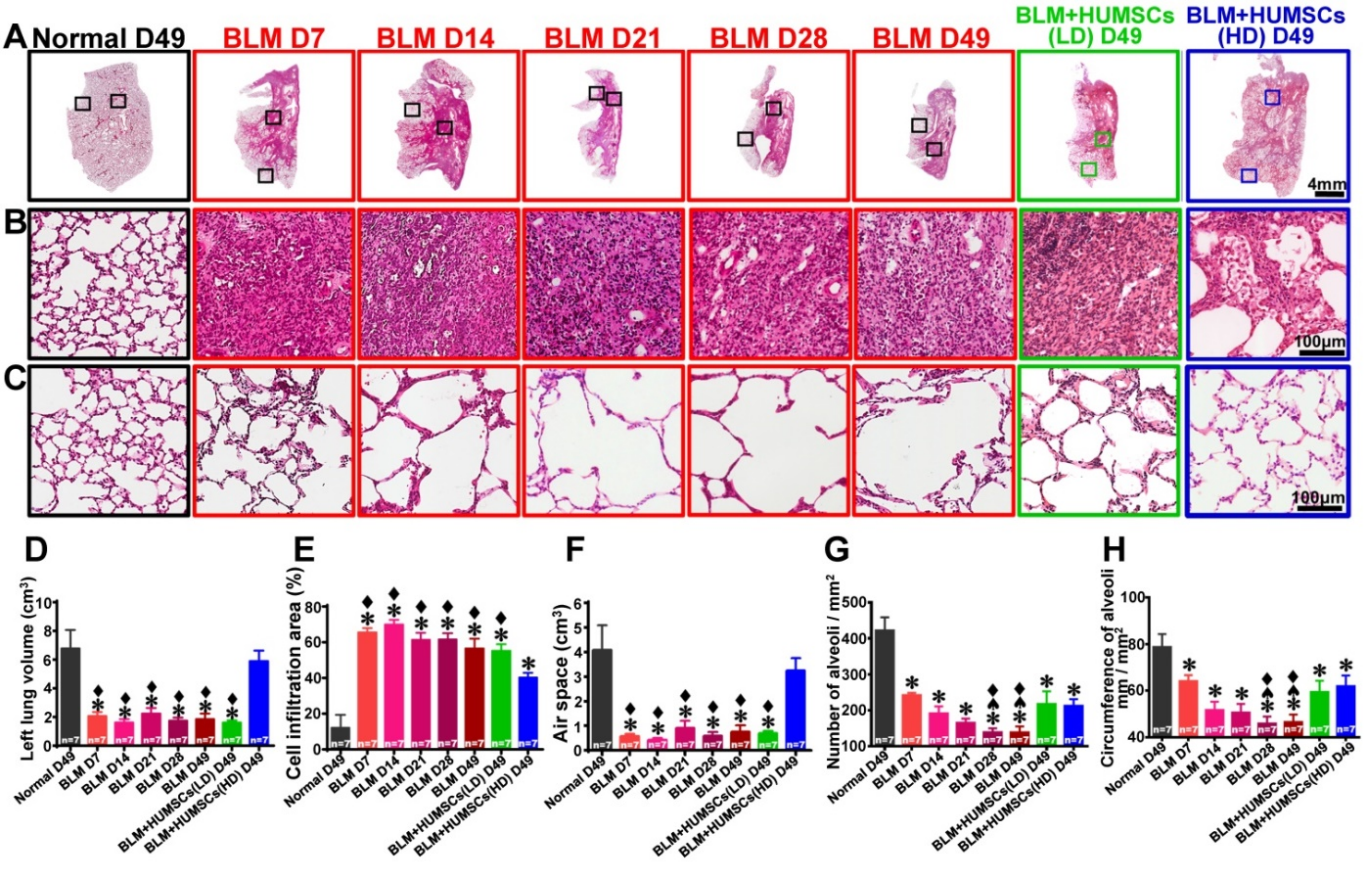
** HUMSC transplantation repaired alveolar structures in PF rats.** The left lung tissue slices from each group were stained with H&E (A). (B) and (C) are enlarged images from the central and peripheral regions, respectively. The results revealed a large cell infiltration in the center areas of the left lungs after BLM injection. Transplantation of high doses of HUMSCs ameliorated cell infiltration conditions in the central areas of the left lungs (B). The total left lung volume was quantified by summing data from all left lung sections, demonstrating that the transplantation of high doses of HUMSCs substantially increased the total left lung volume (D), raised the left lung air space (E), and effectively reduced cell infiltration areas in the left lungs (F). With a relatively small morphology, the number of alveoli per unit area was relatively high in the peripheral region of the left lung in the Normal group (C). Following BLM, the morphology of the alveoli became larger, and therefore, the number of alveoli per unit area decreased. In the group transplanted with HUMSCs, the alveoli were relatively small in morphology (C). The quantification of the number (G) and the circumference of the alveoli (H) per unit area in the peripheral regions of the left lung indicated that transplantation of HUMSCs effectively increased the number of alveoli and circumference per unit area for gas exchange. n=7 animals per group. ✱* vs* the Normal group, p < 0.05. ♠ *vs* the BLM+HUMSCs (LD) group, p < 0.05. ⧫ *vs* the BLM+HUMSCs (HD) group, p < 0.05.

H&E staining showed that the alveoli were intact, and the connective tissues were mostly present near the bronchus in the Normal group. From Days 7 to 49 after BLM injection, alveoli were only present in the outer region of the left lungs and substantial cell infiltration was displayed in the central areas. This situation remained until 49 days after BLM injection (Figures 3A- 3C). The total lung volume, air space and cell infiltration area were similar between the BLM+HUMSCs (LD) and BLM groups on Day 49. However, the left lung volume and the air space in the BLM+HUMSCs (HD) group were similar to those in the Normal group (Figures 3D- 3F).

The alveoli in the peripheries were smaller in size in the Normal group; therefore, the number of alveoli in the unit area was higher, and the total alveolar circumference for gas exchange was longer. The alveolar size in the periphery in the BLM group from Day 7 to Day 49 was significantly larger than that in the Normal group, resulting in a decrease in the total number and alveolar circumference of alveoli in the unit area. The alveolar circumference and number of alveoli in the unit area recovered significantly by Day 49 in the BLM+HUMSCs (LD) and BLM+HUMSCs (HD) groups, indicating that transplantation of HUMSCs improves the efficiency of gas exchange (Figures 3C, 3G, and 3H).

**Figure 4:**
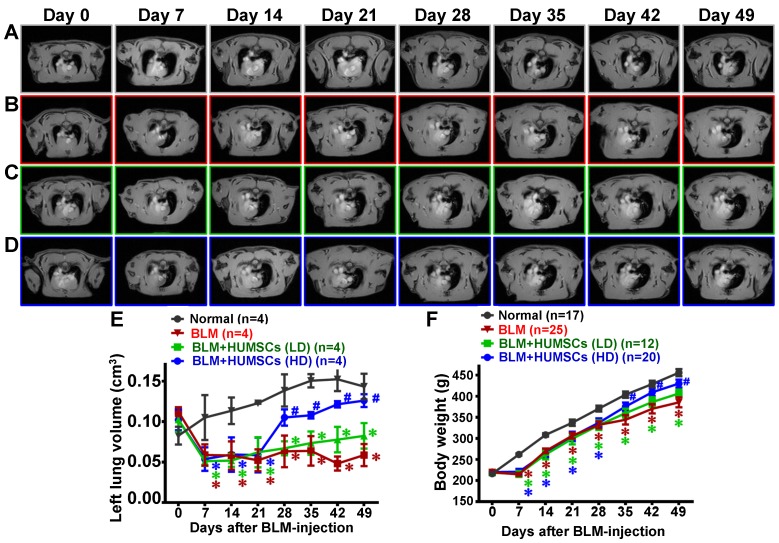
** MRI scans revealed that HUMSC transplantation increased the alveolar volume in the left lungs of rats with PF.** The horizontal image displays the MRI of rats' thoracic cavities from the level of the trachea carina. Black signals in the thoracic cavity represent space occupied by alveoli. L indicates the left side of the body, and R indicates the right. The space occupied by alveoli is clearly seen in both the left and right lungs in the Normal group (A). (B) MRI of the rats' thoracic cavities in the BLM-treated group. On Day 7, the alveolar space was significantly reduced in the left lung, and white consolidated tissues appeared. On Day 14, the alveolar volume in the left lungs was almost completely lost and had become occupied by consolidated tissue, which was sustained until Day 49. MRI of the thoracic cavities in the BLM+HUMSCs (LD) (C) and BLM+HUMSCs (HD) (D) groups is presented. Summing the black alveolar spaces in five MRI scans for each rat indicated that the alveolar volume was significantly reduced following BLM damage. Transplantation of high doses of HUMSCs effectively increased the alveolar volume (E) (n=4 animals per group). The body weights of the rats in each group were documented for 7 weeks following Day 0, when the BLM had not yet been administered. The results indicated that the transplantation of high doses of HUMSCs improved the body weight of rats with PF (F). The animal number per group is shown in F. ✱ *vs* the Normal group at the same time, p < 0.05. **#** *vs* the BLM group at the same time, p < 0.05.

In the horizontal MRI at the level of the trachea carina, the black signals occupying the thoracic cavity are the symbols of the alveoli, whereas the white signals represent consolidated tissues (Figure 4A- 4D and Supplemental Figures 3- 6). Alveoli existed in both lungs of the Normal group, and black signals were observed predominantly from Day 0 to 49 (Figure 4A). White signals appeared in the left lungs because of the inflammatory responses and cell infiltration that occurred on Day 7. The alveolar volume in the left lungs was reduced significantly, and the volume of consolidated tissue was increased on Day 14; this condition persisted up to Day 49 (Figures 4B and 4E). The alveolar volume in the BLM+HUMSCs (LD) group was similar to that in the BLM group from Days 28 to 49 (Figures 4C and 4E). However, the alveolar volume showed no significant difference between the BLM+HUMSCs (HD) and the Normal group from Days 28 to 49 (Figures 4D and 4E).

The body weight in the Normal group increased over time. The weight in the BLM group remained unchanged at Day 7 and was significantly lower than that in the Normal group. The weight trend in the BLM+HUMSCs (HD) showed a marked recovery on Day 35 (Figure 4F).

### Transplantation of HUMSCs improved the respiratory function in rats with PF

**Figure 5:**
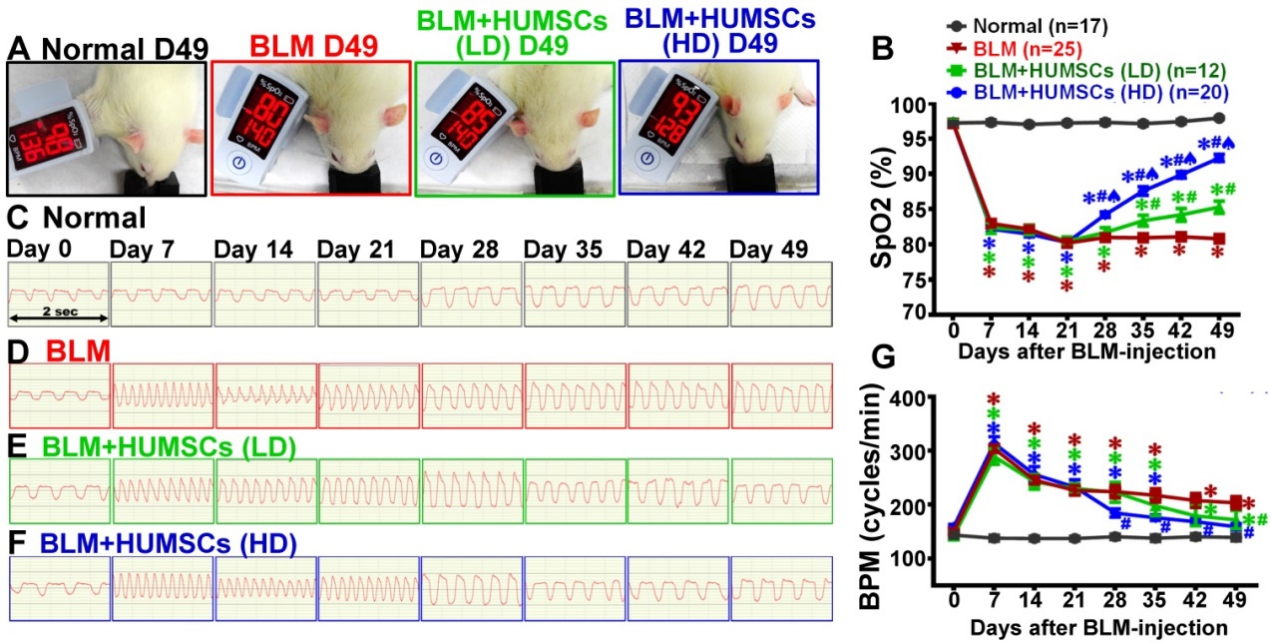
** HUMSC transplantation improved pulmonary function in rats with PF.** The rats in each group were examined for arterial blood oxygen saturation (SpO_2_) in their forelimbs. The photographs reveal that the SpO_2_ on Day 49 was 98% in the Normal group, 80% in the BLM group, 85% in the BLM+HUMSCs (LD) group, and 93% in the BLM+HUMSCs (HD) group (A). The graph demonstrates that the SpO_2_ significantly decreased on Day 7 and this decrease was sustained until Day 49 after BLM injection. Transplantation of HUMSCs helped to increase the SpO_2_ (B). Respiratory rates were recorded weekly for each group. The respiratory frequency was captured within 2 s from Day 0 to 49 in the Normal group (C), BLM-treated group (D), BLM+HUMSCs (LD) group (E) and BLM+HUMSCs (HD) group (F). The quantitative results revealed that the respiratory rate had significantly increased on Day 7, whereas the transplantation of stem cells helped to mitigate the respiratory rate (G). The animal number per group is shown in the figure. ✱ *vs* the Normal group at the same time, p < 0.05. **#** *vs* the BLM at the same time, p < 0.05. ♠ *vs* the BLM+HUMSCs(LD) at the same time, p < 0.05.

A pulse oximeter was used to analyze arterial oxygen saturation (SpO_2_) to evaluate gas exchange. The mean (± SEM) SpO_2_ remained at 97.2% (± 0.8%) on Day 49 in the Normal group. In the BLM group, the SpO_2_ declined to 82.9% (± 3.2%) on Day 7 and was maintained at 80% (± 2.7%) until Day 49, which was significantly lower than that in the Normal group (Figures 5A and 5B; Supplemental Figure 8A).

The SpO_2_ was elevated on Day 35 and persisted up to 85.3% (± 3.0%) on Day 49 in the BLM+HUMSCs (LD) group; the levels of SpO_2_ from Days 35 to 49 in the BLM+HUMSCs (LD) group were higher than those of the BLM group but were still lower than those in the Normal group. The SpO_2_ for BLM+HUMSCs (HD) presented a significant increase on Day 28 and was maintained at 92.3% (± 2.3%) on Day 49, displaying a considerable improvement compared to that in the BLM and BLM+HUMSCs (LD) groups. However, the SpO_2_ was still lower than that in the Normal group (Figures 5A and 5B; Supplemental Figure 8A).

Breaths per minute (BPM) were counted to estimate lung function (Figures 5C- 5G) (Supplemental Figures 7 and 8B). Respiratory rates remained stable in the Normal group (Figures 5C and 5G). The respiratory rates were significantly increased on Day 7 and persisted up to Day 49 after BLM injection (Figures 5D and 5G; Supplemental Figures 7 and 8B). The trend of respiratory rates in the BLM+HUMSCs (LD) and BLM+HUMSCs (HD) groups from Day 7 to Day 21 were similar to those of the BLM group. The respiratory rates were decreased significantly from Day 42 in the BLM+HUMSCs (LD) and from Day 28 in BLM+HUMSCs (HD) (Figures 5E- 5G) (Supplemental Figures 7 and 8B). Thus, the transplantation of HUMSCs enhances respiratory function in rats with PF and thereby mitigates the rapid respiratory rate.

### Transplanted HUMSCs survived in the left lung and did not differentiate into alveolar epithelial cells and vascular endothelial cells

**Figure 6:**
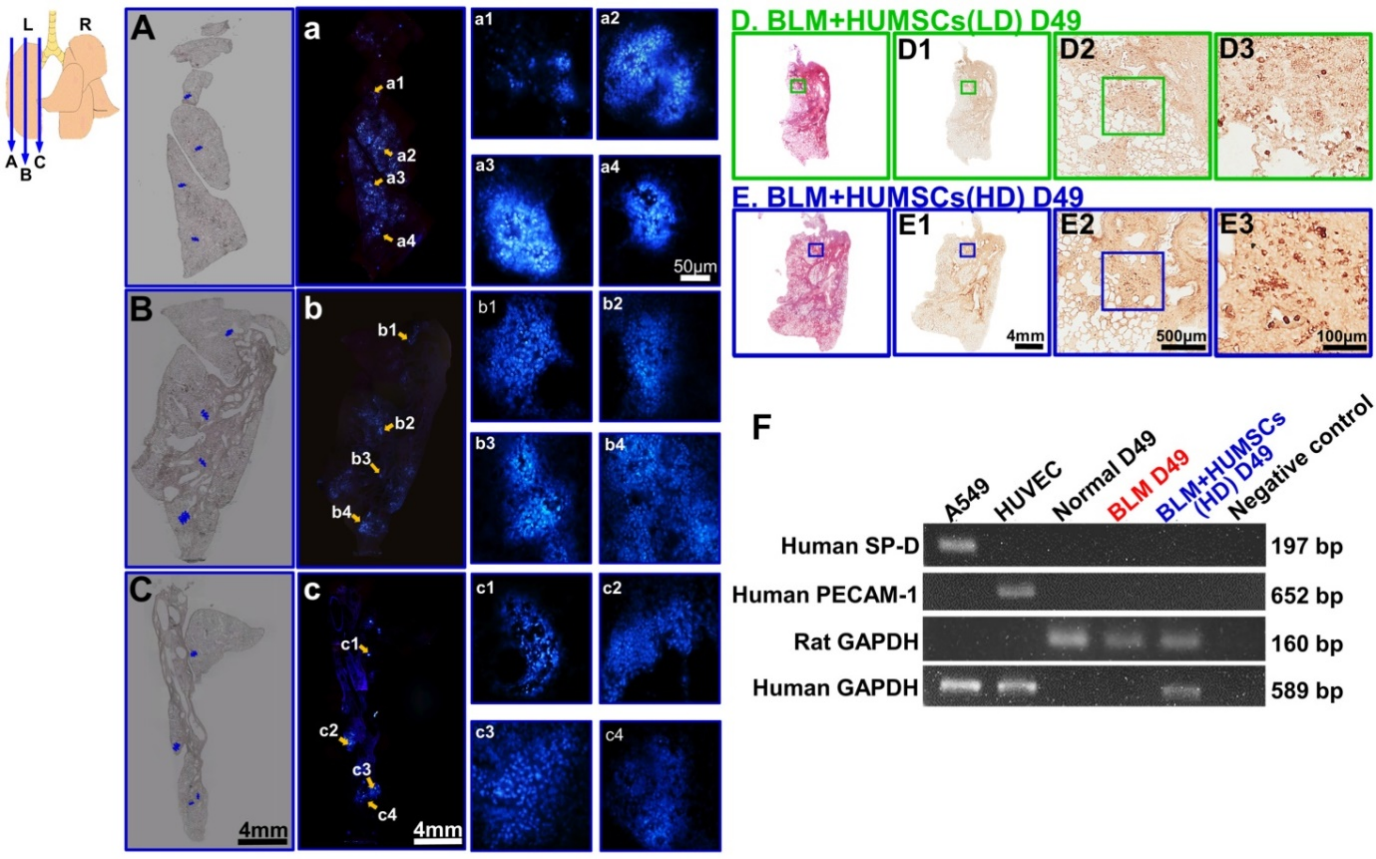
** HUMSCs survived and were scattered in the left lungs of rats with PF without differentiating into alveolar epithelial cells.** HUMSCs cultured *in vitro* were treated with bisbenzimide for 48 h for nuclear labeling (blue). They were subsequently transplanted into the trachea of rats 21 days after BLM injection. At 4 weeks after transplantation, serial cryosectioning was performed. A substantial number of live HUMSCs were found scattered in the lateral (A, a, a1- a4), intermediate (B, b, b1- b4), and inner (close to the hilum; C, c, c1- c4) parts of the lungs in the BLM+HUMSCs (HD) group. The upper-left image represents the locations of the slices. A, B and C are phase images corresponding to different locations in the left lungs; a, b and c are fluorescence images of A, B and C, respectively; and a1- a4, b1- b4, and c1- c4 are magnified images of different regions of a, b and c, respectively. Subsequently, immunohistochemistry was performed with an anti-human nuclei antibody to label HUMSCs. From low to high magnifications, numerous HUMSCs were observed to be scattered in the left lungs of both BLM+HUMSCs (LD) (D, D1- D3) and BLM+HUMSCs (HD) groups (E, E1- E3). The left lung tissues from the Normal, BLM-treated, and BLM+HUMSCs (HD) groups on Day 49 were subjected to RT-PCR to identify the cDNA of human surfactant protein D (SP-D), and human platelet endothelial cell adhesion molecule (PECAM-1) was used to examine whether HUMSCs had differentiated into human alveolar or vascular endothelial cells (F). The differentiation of HUMSCs was examined using RT-PCR. A549 is a human adenocarcinoma cell line and was used as a positive control for human SP-D and human GAPDH. Human umbilical vein endothelial cells were applied as positive controls for human PECAM-1 and human GAPDH. The results indicated that HUMSCs located in the rats' left lungs do not differentiate into alveolar epithelial or vascular endothelial cells.

A substantial number of live HUMSCs labeled with bisbenzimide (blue fluorescence) were observed scattered over the lateral (Figure 6A, a, a1- a4), central (Figure 6B, b, b1- b4) and inner (close to the hilum; Figure 6C, c, c1- c4) parts of the left lung. The results reveal that the HUMSCs survived and distributed throughout the left lungs of the rats.

Additionally, anti-human specific nuclei antigen antibody was used to label HUMSCs. In the left lungs of both the BLM+HUMSCs (LD) and BLM+HUMSCs (HD) groups, HUMSCs were found in the connective tissues and scattered among the alveoli (Figures 6D, 6D1- 6D3, 6E, and 6E1- 6E3). Furthermore, complementary DNA (cDNA) of human GAPDH was detected in the rat's lung of BLM+HUMSCs(HD) group on Day 49 (Figure 6F). These results demonstrated the existence of HUMSCs in rat's lung.

Complementary DNA of human surfactant protein D (SP-D) and human platelet endothelial cell adhesion molecule (PECAM-1) identification were used to examine whether HUMSCs had differentiated into alveolar or endothelial cells. The expression of human glyceraldehyde-3-phosphate dehydrogenase (GAPDH) was detected in the lungs of the BLM+HUMSCs (HD) group. However, the expression of human SP-D or PECAM-1 was not observed in the BLM+HUMSCs (HD) group, indicating that the HUMSCs did not differentiate into alveolar or vascular endothelial cells (Figure 6F). In addition, immunohistostaining of rabbit anti- human Podoplanin gp36 antibody for left lung was used to examine whether HUMSCs had differentiated into human alveolar type I cells. The positive immunostaining was hardly found in the lung of the BLM+HUMSCs (HD) group (data not shown).

### HUMSC transplantation triggers the activation of macrophages and facilitates the synthesis of MMP-9 in macrophages to degrade collagen

**Figure 7:**
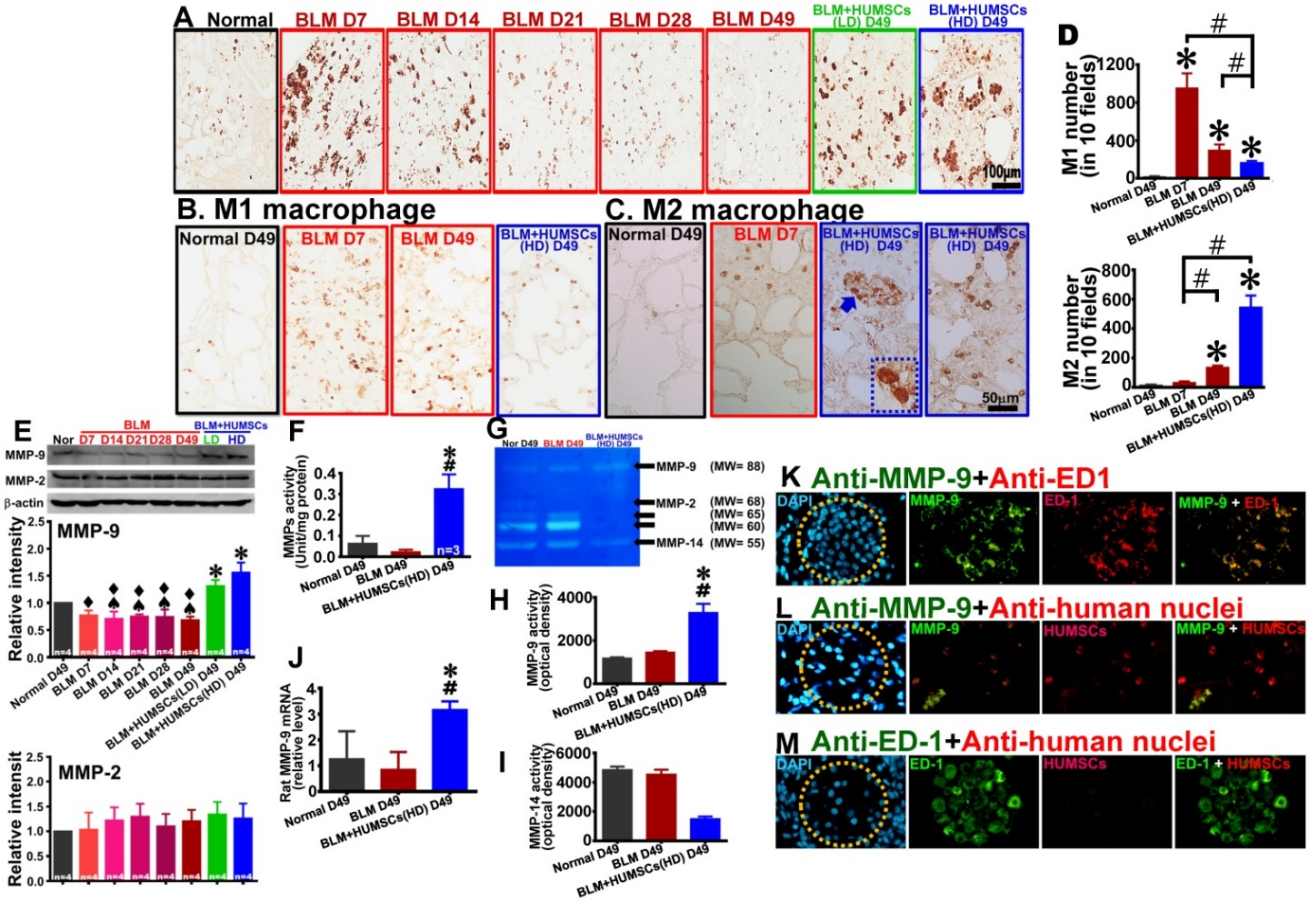
** Transplantation of HUMSCs stimulated macrophage synthesis of MMP-9 in the left lungs of rats with PF.** (A) Left lung sections were subjected to immunohistochemistry with an anti-ED1 antibody for macrophage labeling. On Days 7 and 14, the macrophages in the left lung were extensively activated. However, from Day 21 to 49, the number significantly decreased. Transplantation of HUMSCs stimulated the activation of macrophages. Immunohistostaining of anti-iNOS (B) and anti-CD163 (C) were performed to detect M1 and M2 macrophages in the lung respectively. (D) The quantitative results showed that M2 macrophages significantly increased, whereas M1 macrophages statistically decreased in the group of BLM+HUMSCs (HD) on Day 49. Magnified image of the boxed area (arrow) demonstrated that highly active M2 macrophages were present in the alveoli. (E) The contents of MMP-9 and MMP-2 in the rats' left lungs were detected through western blotting. The quantitative results indicated that MMP-9 significantly increased following the transplantation of HUMSCs, whereas no significant alteration was found for MMP-2. (F) The proteolytic activities of total MMPs in the left lung tissue lysates were measured by the gelatin fluorometric assay. (G) Representative image of gelatin zymography showing MMPs activity in left lung tissue lysate. (H and I) Gelatin zymography results showed that MMP-9 activity was significantly increased, whereas MMP-14 or other MMPs was significantly decreased in the group of BLM+HUMSCs (HD) at Day 49. (J) Quantitate real-time PCR analysis of relative expression of rat MMP-9 mRNA in lungs (n = 3/group). To further explore whether increases in MMP-9 came from HUMSCs or macrophages, the left lung tissue sections from the BLM+HUMSCs (HD) group on Day 49 were subjected to double-staining with either (K) anti-MMP-9 (green) and anti-ED1 (red) antibodies, (L) anti-MMP-9 (green) and anti-human nuclei (red) antibodies, or (M) anti-ED1 (green) and anti-human nuclei (red) antibodies. The results indicated that MMP-9 was predominantly synthesized and secreted by macrophages. Additionally, transplanted HUMSCs did not differentiate into macrophages or synthesize MMP-9. Dotted circles indicate alveoli. n= 4 animals per group. ✱* vs* the Normal group, p < 0.05. **#** *vs* the BLM group, p < 0.05. ♠ *vs* the BLM+HUMSCs (LD) group, p < 0.05. ⧫ *vs* the BLM+HUMSCs (HD) group, p < 0.05.

Anti-ED1 antibody was used to identify macrophages. Macrophages that were a relatively small size and few in number were found in the Normal group. Following BLM injection, macrophages were activated, with an increased number and a larger size on Days 7 and 14. However, the activated macrophages decreased from Day 21 to 49. In the BLM+HUMSCs (LD) and BLM+HUMSCs (HD) groups, the activated macrophages were scattered in the connective tissues and showed an increase in amount and size (Figure 7A). In order to discriminate M1 macrophage from M2 macrophage, the immunostaining of anti-iNOS for M1 macrophage and anti-CD163 for M2 macrophage were performed respectively. The number of M1 macrophage boosted on Day 7 and declined on Day 49 after BLM. Compared with the BLM group, M1 macrophage content substantially decreased in the BLM+HUMSCs (HD) group on Day 49 (Figures 7B and 7D). The number of active M2 macrophage was higher significantly in the BLM group than that in Normal group on Day 49, but lower significantly than that in the BLM+HUMSCs (HD) group. In the group of BLM+HUMSCs (HD), a lot of M2 macrophages were present in the fibrotic areas, and a great quantity of M2 macrophages with dense vesicles in the cytoplasma were observed within the alveolar-like structures (Figures 7C and 7D).

The results of western blotting revealed that the expression of matrix metallopeptidase 9 (MMP-9) was considerably increased in the BLM+HUMSCs (LD) and BLM+HUMSCs (HD) groups on Day 49, whereas no significant difference was observed for MMP-2 (Figure 7E). In order to assess the activity of total MMPs after BLM injury and HUMSCs transplantation, the gelatinolytic activity in the left lung was analyzed. The results showed that gelatinolytic activities of total MMPs in the group of BLM+HUMSCs (HD) displayed stronger than those in the Normal and BLM groups on Day 49 (Figure 7F). In order to further identify the individual MMP involved, zymography analysis was performed. The results demonstrated that the expression of active MMP-9 (MW=88 kD) in the BLM+HUMSCs (HD) group was higher than those in the Normal and BLM groups on Day 49 (Figures 7G and 7H). The active MMP-2 (MW=68 kD) and other MMPs (MW=65 and 60 kD) presented undetectable expressions in the BLM+HUMSCs (HD) (Figure 7G). Notably, active MMP-14 (MW=55 kD) was lower significantly in BLM+HUMSCs (HD) than those in Normal and BLM groups (Figures 7G and 7I). To further confirm the origin of increase of MMP-9 activity, real-time RT-PCR was used to quantify the level of human and rat MMP-9 mRNA in left lung. Human MMP-9 mRNA did not been detected in the BLM+HUMSCs (HD) group (data not shown), however, rat MMP-9 mRNA displayed significant elevation in the BLM+HUMSCs (HD) group (Figure 7J).

To further explore whether increases in MMP-9 came from HUMSCs or host macrophages, double-fluorescence staining was conducted with anti-MMP-9/anti-ED1 and anti-MMP-9/anti-human nuclei antibodies. Numerous cells were stained with MMP-9, most of which were synthesized by macrophages in the BLM+HUMSCs (HD) group (Figure 7K). However, co-localization of MMP-9 and HUMSCs was barely observed (Figure 7L). Furthermore, ED1-positive macrophages and HUMSCs were barely colocalized (Figure 7M). This finding suggests that transplantation of HUMSCs stimulates the activation of autologous macrophages to synthesize MMP-9 and thereby assist with the degradation of collagen.

### HUMSC transplantation decreases lung inflammation, reduces the activation of myofibroblasts and enhances TLR-4 expression

**Figure 8:**
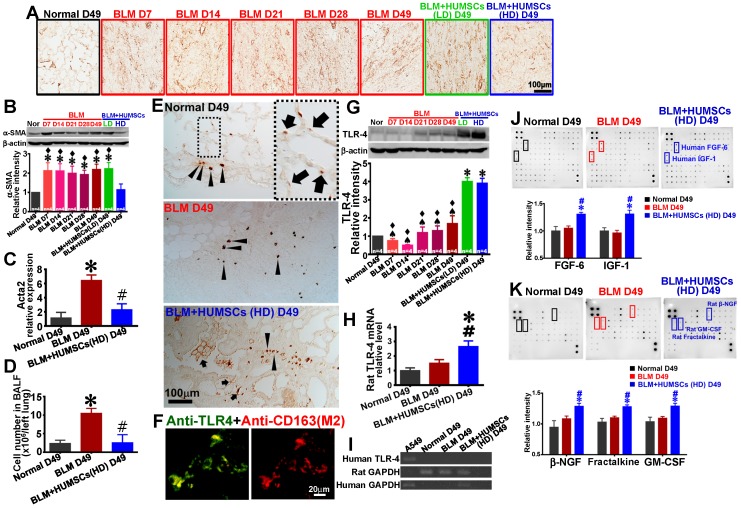
** Transplantation of HUMSCs reduced the activation of fibroblasts and promoted the expression of TLR-4 in the left lungs of rats with PF.** (A) Left lung sections were labeled with anti-α-SMA antibody to indicate activated fibroblasts in the left lungs of each group. (B) Western blot images represent the contents of α-SMA in the rats' left lungs. Quantitative results of α-SMA from western blotting are shown. The results indicated that activated fibroblasts significantly increased from Day 7 to 49. The activated fibroblasts significantly decreased after the transplantation of high doses of HUMSCs. (C) Rat *Acta*2 mRNA were extracted from left lung suspensions and analyzed by quantitative RT-PCR, demonstrating that a significant decrease in acta2 expression in the BLM+HUMSCs (HD) group on Day 49. (D) Cell number in bronchoalveolar lavage fluid (BALF) of the BLM+HUMSCs (HD) group reduced significantly compared with that in the BLM group on Day 49. (E) The immunohistostaining of anti-TLR-4 showed that some TLR-4 positive cells were localized in connective tissue (arrow heads), however, some in the alveoli (arrows). (F) To further explore whether TLR-4 expression was localized in M2 macrophage, the left lung tissue sections from the BLM+HUMSCs (HD) group on Day 49 were subjected to double-staining with anti-TLR-4 (green) and anti-CD163 (red) antibodies. (G) The contents of TLR-4 in the rats' left lungs were detected through western blotting. The quantitative results indicated that TLR-4 significantly increased following the transplantation of HUMSCs. (H) Quantitative analysis of real-time RT-PCR of rat TLR-4 mRNA in the left lung tissue lysate demonstrated that rat TLR-4 mRNA was increased in the BLM+HUMSCs (HD) group (n = 3/group). (I) The result of RT-PCR of human TLR-4 revealed that human TLR-4 was only found in cell lysate of A549 cell line, but not in the lungs of all groups. (J) Left lung samples from the Normal, BLM, and BLM+HUMSCs (HD) groups on Day 49 were subjected to Human Cytokine Antibody Array analysis. The results suggested that the transplantation of HUMSCs increased human FGF-6 and IGF-1 concentrations in rats with pulmonary fibrosis. (K) Analyses were conducted using Rat Cytokine Antibody Array. The results indicated that the transplantation of HUMSCs stimulated rats with pulmonary fibrosis to produce higher amounts of β-NGF, fractalkine, and GM-CSF in their left lungs. ✱* vs* the Normal group, p < 0.05. # *vs* the BLM group, p < 0.05. ♠ *vs* the BLM+HUMSCs (LD) group, p < 0.05. ⧫ *vs* the BLM+HUMSCs (HD) group, p < 0.05.

Anti-α-SMA antibody was used to identify myofibroblasts. Few myofibroblasts were discovered in the Normal group. The expression of α-SMA was considerably increased in the BLM group, whereas the expression was markedly reduced in the BLM+HUMSCs (HD) group (Figures 8A and 8B). Simultaneously, real-time RT-PCR was used to quantify the level of rat* Acta2* mRNA in fresh left lung. The relative expression of rat *Acta2* of the BLM group increased significantly compared to those of the Normal group and the BLM+HUMSCs (HD) group on Day 49 (Figure 8C).

Cell number measured in BALF was used to estimate the amplitude of pulmonary inflammation. Cell number in BALF of BLM+HUMSCs (HD) presented a significant decrease compared with that of BLM group on Day 49, displaying a considerable improvement in lung inflammation after BLM injury (Figure 8D).

TLR-4 immunohistostaining showed that few TLR-4 positive cells were present near the bronchi in the Normal group, suggesting they were pulmonary macrophages. At the same time, TLR-4 positive spots were existed in the alveoli, suggesting they were localized in AEC2s (Figure 8E). An increased number of TLR-4 positive cell within fibrotic area was observed in the BLM group on Day 49 (Figure 8E). In the group of BLM+HUMSCs (HD), a large number of cell staining positively for TLR-4 were found within fibrotic area, moreover, robustly stained spots were discovered around the alveolar circumference (Figure 8E). From the result of double-fluorescence staining of anti-TLR-4 and anti-CD163 revealed that most of TLR-4 positive cells were localized in the M2 macrophages in the connective tissue in the BLM+HUMSCs (HD) group (Figure 8F). Western blotting for TLR-4 showed that the expression of TLR-4 was significantly amplified in the BLM+HUMSCs (LD) and BLM+HUMSCs (HD) groups, indicating that transplantation of HUMSCs promotes the restoration of alveolar epithelial cells (Figure 8G). To further confirm the origin of increase of TLR-4, real-time RT-PCR was used to quantify the level of human and rat TLR-4 mRNA in left lung. Rat TLR-4 mRNA displayed significant higher in the BLM+HUMSCs (HD) group than those in the Normal and BLM groups (Figure 8H), however, human TLR-4 mRNA did not been detected in the BLM+HUMSCs (HD) group on Day 49 (Figure 8I).

### Transplanted HUMSCs released cytokines that facilitated the recovery of lungs in rats with PF

To investigate whether HUMSCs transplanted into rat lungs secrete human cytokines and whether they alter rats' autologous cytokine profile, Human Cytokine Antibody Array and Rat Cytokine Antibody Array were used to examine the alterations of 174 human cytokines and 34 rat cytokines, respectively. Substantial amounts of human FGF-6 and IGF-1 existed in the BLM+HUMSCs (HD) group (Figure 8J). Furthermore, the transplantation of high doses of HUMSCs also stimulated the increased production of rat β-NGF, fractalkine, and GM-CSF (Figure 8K).

### HUMSCs released HA and HUMSCs promoted the TLR-4 expression of alveolar epithelial cells in *ex vivo* coculture experiments

**Figure 9:**
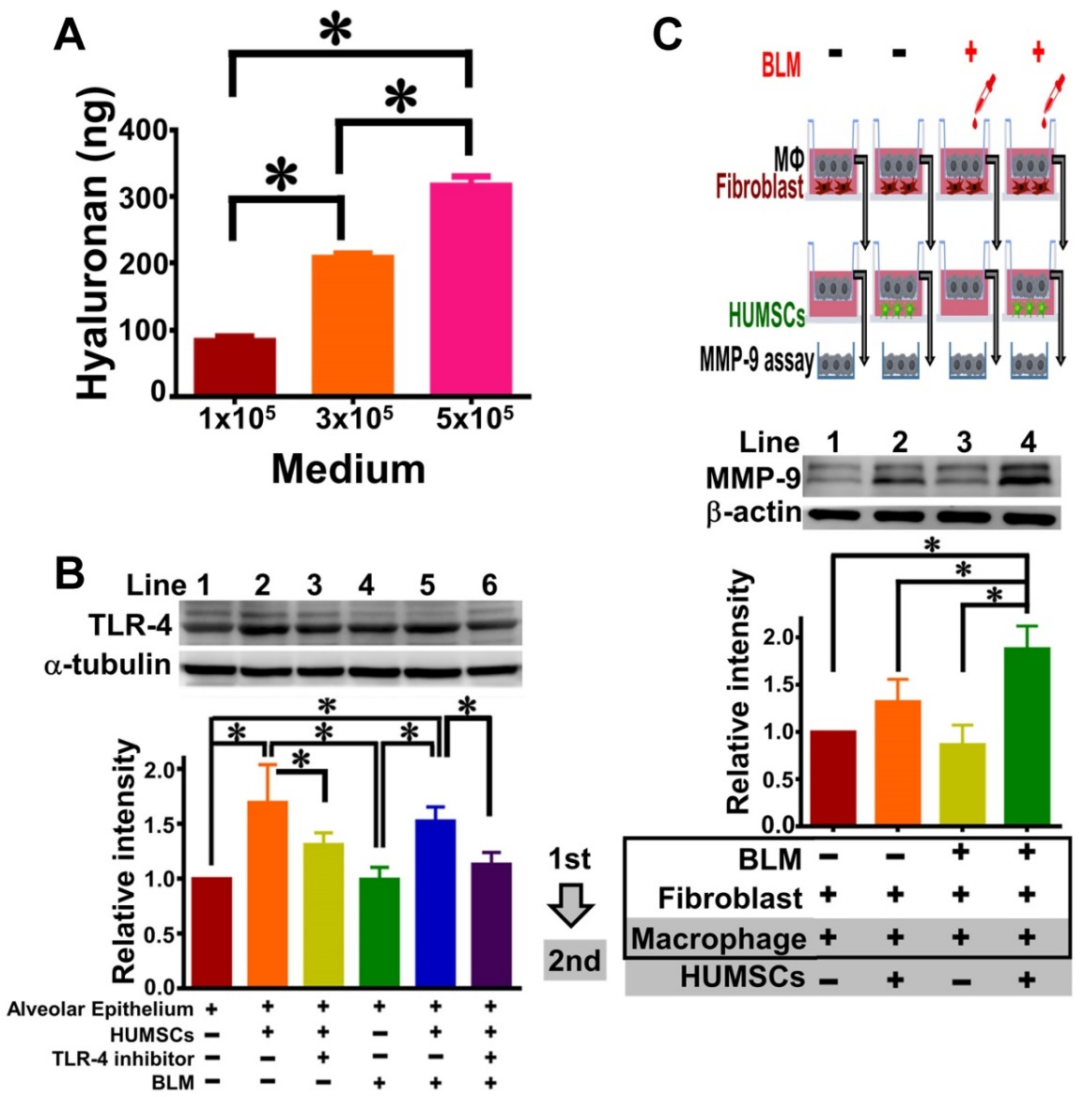
** HUMSCs stimulate macrophages to synthesize MMP-9 and release HA to trigger alveolar epithelial expression of TLR-4 in the coculture system.** Western blot images represent the content of MMP-9 in alveolar macrophages in the coculture of pulmonary macrophages, fibroblasts and HUMSCs. The quantitative results indicate that HUMSCs can enhance MMP-9 synthesis in macrophages after BLM treatment (A). The quantity of HA released into the media by HUMSCs is cell amount-dependent (B). Western blot images from the coculture of alveolar cells and HUMSCs showed that HUMSCs resulted in an increase in TLR-4 expression in the alveolar cells, irrespective of BLM (C). n=4. ✱, p < 0.05.

The quantity of HA released from HUMSCs was evaluated by Hyaluronan Quantikine ELISA (R&D Systems). Mean (± SEM) values of 85.91 (± 8.10), 209.96 (± 8.56) and 318.13 (± 20.97) ng of HA were measured from 10 mL culture medium with 10^5^, 3×10^5^, and 5×10^5^ HUMSC density, respectively (Figure 9A).

To evaluate the effect of HUMSCs towards alveolar epithelial cells, specifically on TLR-4, HUMSCs (upper) and alveolar epithelial cells (lower) were cocultured. The results of immunoblotting from alveolar epithelial cells showed that (1) the expression level of TLR-4 significantly increased (Figure 9B, Lane 2), a specific regulatory inhibitor of TLR-4 can prevent this enhancement effect (Lane 3). (2) In the system of alveolar cells cocultured with HUMSCs under BLM induction, the level of cellular TLR-4 in alveolar cells increased statistically (Lane 5). In contrast, in the presence of a TLR-4 inhibitor, no apparent increase in TLR-4 was observed in alveolar cells (Lane 6), despite coculture with HUMSCs. This result shows that HUMSCs promotes TLR-4 expression in alveolar epithelial cells.

### HUMSCs promote macrophages to synthesize MMP-9 in the coculture of pulmonary macrophages, fibroblasts and HUMSCs

To investigate whether HUMSCs promote the expression of MMP-9 in pulmonary macrophages, the coculture of pulmonary macrophages and fibroblasts and the coculture of pulmonary macrophages and HUMSCs were performed in sequence. The immunoblot of MMP-9 in macrophages without BLM induction was set as relative intensity 1 (Lane 1, Figure 9C); however, the BLM-induced macrophages showed a difference in MMP-9 of only 0.86±0.41-fold, indicating no significant alteration in the MMP-9 level of macrophages with or without BLM (Lane 3, p > 0.05). After transferring the macrophages to the second coculture with HUMSCs, the MMP-9 level slightly increased 1.32 ± 0.47-fold in macrophages (Lane 2). BLM-treated macrophages were then cocultured with HUMSCs; the results showed significant MMP-9 expression (1.88 ± 0.48, p < 0.05, Lane 4) in BLM-induced macrophages.

## Discussion

In this study, HUMSCs were xenografted into the lungs of rats with PF, and our results suggest that the transplanted HUMSCs can survive for a long time and effectively reverse PF.

Many studies have demonstrated that stem cell transplantation in the acute phase of lung injury can reduce or prevent PF 16, 20, 22, 24. However, stem cell transplantation for treating PF is limited by time and dosage. Bone marrow MSCs transplanted immediately after injury were found to differentiate into functional lung cells, whereas stem cells transplanted at later stages were shown to exacerbate PF 16, 50. In practice, most patients attending clinics for respiratory problems have already developed different degrees of PF, rendering therapy with bone marrow MSCs too late after the onset of disease.

In our study, BLM-induced pathological progress in the left lung was represented by the alveoli disappearing and connective tissue-like structures appearing on Day 7. The connective tissues in the central areas became shriveled, and the collagen content markedly increased and reached a plateau from Day 21 to Day 49 after BLM injection. We suggest that lung injury on Day 21 was equal to the later stage of lung damage. In addition, collagen has been shown to markedly increase at 14 days after BLM instillation 20.

Another conclusion about stem cell quantity after intravenously injected low (2×10^4^), medium (10^5^), and high doses (2×10^5^) of human bone marrow MSCs into rats with lung damage is worth mentioning. The results revealed stronger anti-inflammatory responses in the low-dose group compared with those in groups given the medium or high doses 23. In the present study, transplantation of low-dose HUMSCs (5×10^6^) showed unsatisfactory consequences in PF. However, transplantation of high-dose HUMSCs (2.5×10^7^) displayed significant efficacy for PF. Therefore, the number and survival time of transplanted stem cells are the critical factors to reverse PF. We suggest that the different effects may be due to differences in stem cell sources, resulting in diverse cell properties and various outcomes 51, 52. Next, we will examine and compare the efficacy of transplantation of bone marrow MSCs or adipose tissue MSCs in this animal model of BLM-induced one-sided and severe PF.

Because the number of transplanted stem cells is considered as an important issue to regeneration, HUMSCs were implanted into different target-organs depending on the diseases. In our previous studies, HUMSCs survive in the individual organs after being engrafted into the striatum, spinal cord, liver or bone marrow of rats and are difficult to find their existences in other organs 29, 32, 34, 37, 38. In this study HUMSCs were implanted intratracheally to allow most transplanted stem cells migrating into damaged lung. Rare HUMSCs were found in the right lung. Clinically, it is always not recommended to use invasive transplantation methods. Fortunately, the majority of cells administered intravenously are found in the lungs primarily following delivery 53-55. In this study, the amount of high-dose HUMSCs (2.5×10^7^) per rat showed significant improvement for PF, we extrapolate that the number of transplanted cell for human patient is about 2.5×10^9^. We suggest that repeated administrations of HUMSCs by the intravenous route (non-invasive) could be applied for PF patients. Previous studies have shown that HUMSCs can regulate or suppress immune cells, supporting that xeno-transplanted HUMSCs can survive in different organs of SD rats with normal immune system 56-60.

Glasser et al. 61 proposed that the successful resolution of PF involves inhibiting the activation and elimination of fibroblasts, promoting the degradation of the ECM, and stimulating the regeneration and reconstitution of the alveolar epithelium. The present study demonstrated that HUMSC transplantation successfully lessened inflammation, specifically, a reversal of myofibroblast phenotype (anti-α-SMA experiments). As a result, new ECM synthesis and accumulation were significantly diminished and halted. Similar conclusions are supported by previous studies, even when the sources of stem cells are different 16, 20, 24.

Additionally, the transplantation of HUMSCs may also decompose existing collagen by triggering the activation of macrophages and stimulating the synthesis and secretion of MMP-9 from macrophages. The sequential effect is to accelerate the degradation of the existing ECM. A similar finding was obtained by Cabrera et al., who reported that the overexpression of MMP-9 in macrophages attenuates BLM-induced PF 21.

Pharmacological activation of TLR-4 ameliorated inflammation and PF and improved lung function 62. Either deletion of TLR-4 or HA synthase-2 in AEC2s results in impaired renewal capacity and severe fibrosis. Furthermore, a loss in cell surface expression of HA was found in AEC2s isolated from the lungs of patients with severe PF 63. Their main finding was that TLR-4 and HA play critical roles in the regeneration of the alveolar epithelium. Additionally, HA appears to have a modulatory effect on immune cells 43, 44, 64. HA interaction with TLR4 can educate macrophage polarization to an M2-like phenotype 65. HA was synthesized and released from HUMSCs* in vitro* in this study (Figure 9A). We suggest that HA released continuously from transplanted HUMSCs may be one of the reasons why HUMSCs are more advantageous than other MSCs. Moreover, Co-localization of TLR4 and M2 macrophage were found in the group of BLM+HUMSCs (HD). HUMSC transplantation into rats with PF increased the TLR-4 content in the lung from the results of western blotting and TLR-4 immunohistostaining, suggesting that HUMSCs promote the pathway of HA and TLR-4 in both alveolar epitheliums and macrophages. Interestingly, TLR-4 expression increased in WT C57Bl/6 mice on day 7 after BLM 63 and in SD rats on day 21 (in this study) respectively. The discrepancy of timing of TLR-4 raise in these two animals might result from the difference of animal species or the inconsistency of the lung damage. The significant elevation of TLR-4 in lung may be the self-compensation to trigger recovery, eventually, the amount of TLR-4 of alveolar epithelium after BLM might be insufficient to repair injured lung.

In addition to HA, the levels of human FGF-6 and IGF-1 were found to be relatively higher in the left lungs of these rats. Hogan et al. claimed that the FGF family is associated with alveoli development through its role in the process of differentiation from progenitor cells to alveolar epithelial cells 66. IGF-I was indicated as reducing phagocytosis in epithelial cells and therefore helping to diminish its inflammatory reaction 67. Thus, we surmised that HUMSCs transplanted into BLM-treated rat lungs secrete a variety of cytokines that effectively resolve pulmonary fibrosis. We speculated that some other cytokines and growth factors beyond the 174 that we assessed may also stimulate the regeneration of fibrotic lungs. From the results of the rat cytokine array, we suggest the implanted HUMSCs impact and change the host cells, including alveolar cells, fibroblasts and macrophages. We will explore the exact pathway and the interaction between the HUMSCs and host cells in the future. Notably, in response to the corresponding disease types and the organs where they were implanted, transplanted HUMSCs secreted various cytokines under various microenvironments, which facilitated the recovery of damaged tissues. We previously demonstrated that HUMSC transplantation treated ischemic stroke in rats primarily through the cytokines secreted by the HUMSCs, such as NAP-2, angiopoietin-2, BDNF, CXCL-16, and PDGF-AA. Therapeutic effects were achieved because these cytokines promoted the regeneration and protection of endogenous neuronal cells, which ameliorate functional loss after stroke 33. In addition, the transplantation of HUMSCs into completely transected rat spinal cords increased the expressions of NT-3, NAP-2, bFGF, GITR, and VEGF-R3, which may facilitate spinal cord regeneration 34. Moreover, by secreting a substantial amount of prolactin, LIF, and CTACK, HUMSCs implanted into fibrotic livers successfully repaired liver damage 37.

**Figure 10:**
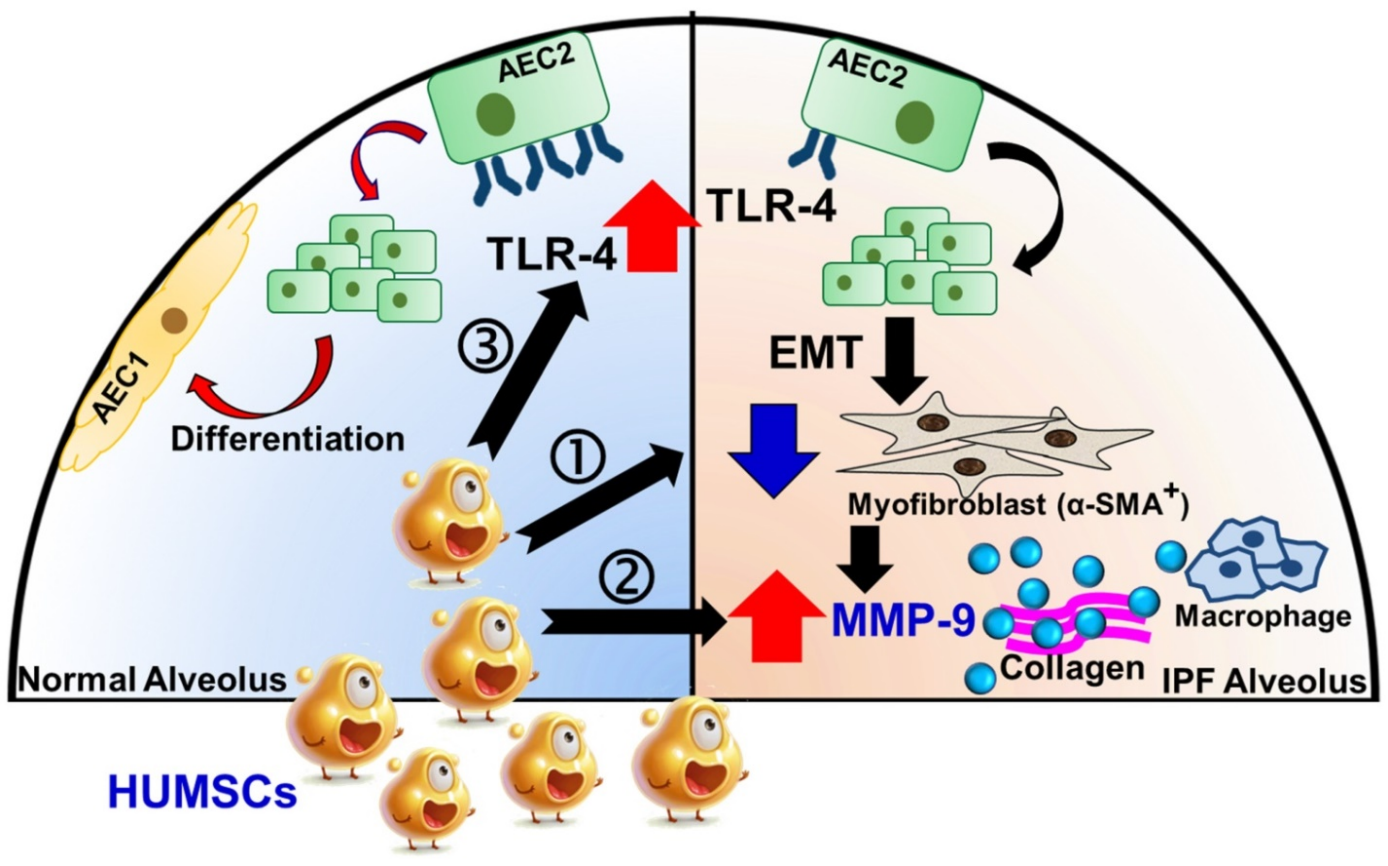
Potential mechanisms of HUMSCs in the successful resolution of pulmonary fibrosis.

In conclusion, xenografted HUMSCs did not differentiate into functional lung cells but secreted a variety of cytokines and HA that effectively reversed PF. Three underlying therapeutic mechanisms exist. First, HUMSCs inhibited inflammation, reduced myofibroblast activity, and prevented more collagen synthesis. Second, HUMSCs activated the host's macrophages to synthesize MMP-9, which degraded the existing collagen. Third, HUMSCs promoted the expression of TLR-4 in the host's alveolar epithelial cells and triggered the signal of HA-TLR-4 for lung regeneration (Figure 10). We hope that HUMSC transplantation provides a new therapeutic strategy for patients with PF.

## Supplementary Material

Supplementary figures and tables.
