# Supplementary material for: Reversal of bleomycin-induced rat pulmonary fibrosis by a xenograft of human umbilical mesenchymal stem cells from Wharton's jelly
Source: Theranostics. 2019 Sep 17;9(22):6646–64. doi: 10.7150/thno.33741 (PMC6771241; doi:10.7150/thno.33741)
Supplement: Supplementary file 1 — Supplementary figures and tables. [file thnov09p6646s1.pdf]

Supplemental Figure 1

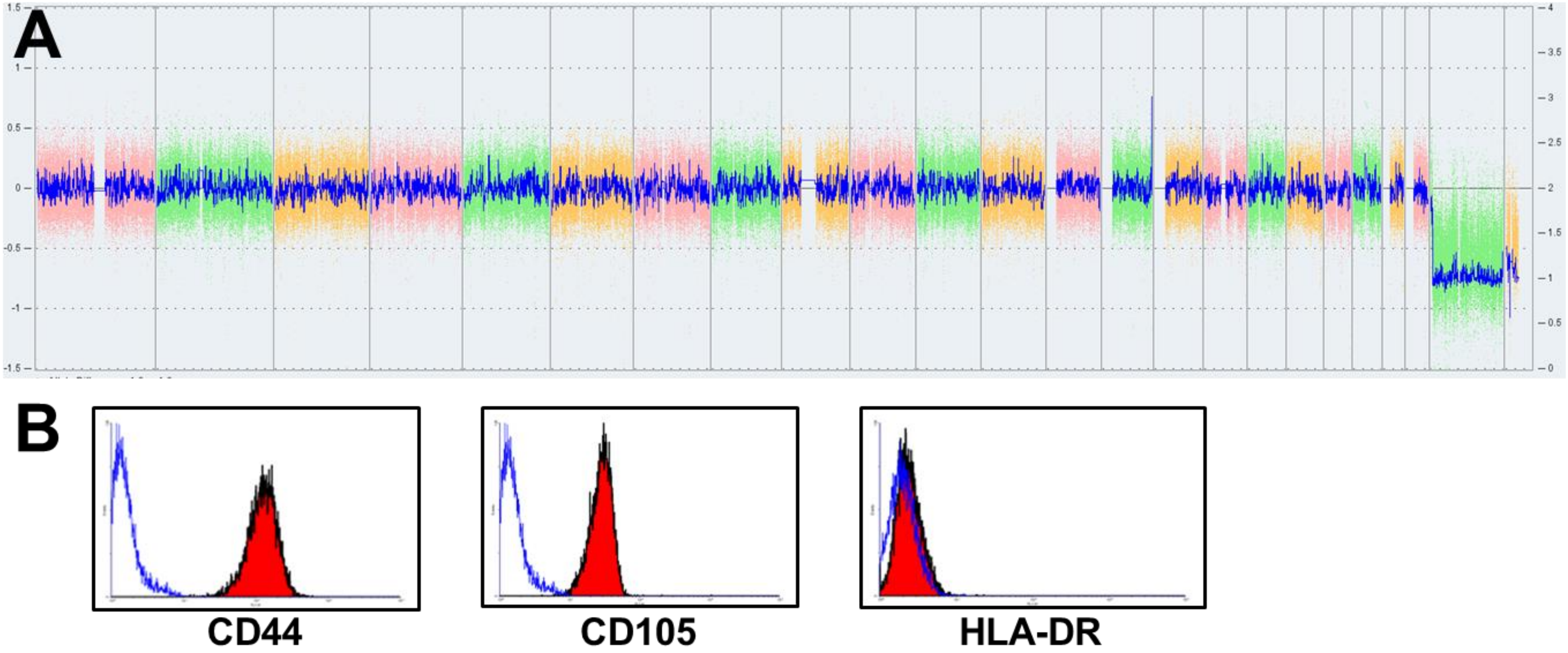

Supplemental Figure 1. Chromosomal karyotyping and surface markers of HUMSCs *in vitro*.

(A) To analyze the copy number of 23 chromosomes of HUMSCs *in vitro* (passage 10th), the CytoScan 750K Array (Affymetrix) was used to screen the chromosomal karyotype. The X-axis represents the chromosome number (No. 1-22, XY), the right Y-axis represents the copy number of chromosome (0, 1, 2, or 3), and the blue line is the copy number of chromosomes performed by the company of Genetics Generation Advancement. The result indicated that the chromosomes No. 1- 22 are two sets and sex chromosome are X+Y. (B) Flow cytometry analyses of surface markers of HUMSCs *in vitro*. HUMSCs were cultured for 10 passages and then labeled with CD44, CD105 and HLA-DR antibodies. White areas represent negative controls and red areas represent the specific binding for indicated antigens. The results revealed that HUMSCs transplanted into rats were positive for CD44 and CD105 but negative for HLA-DR.

Supplemental Figure 2

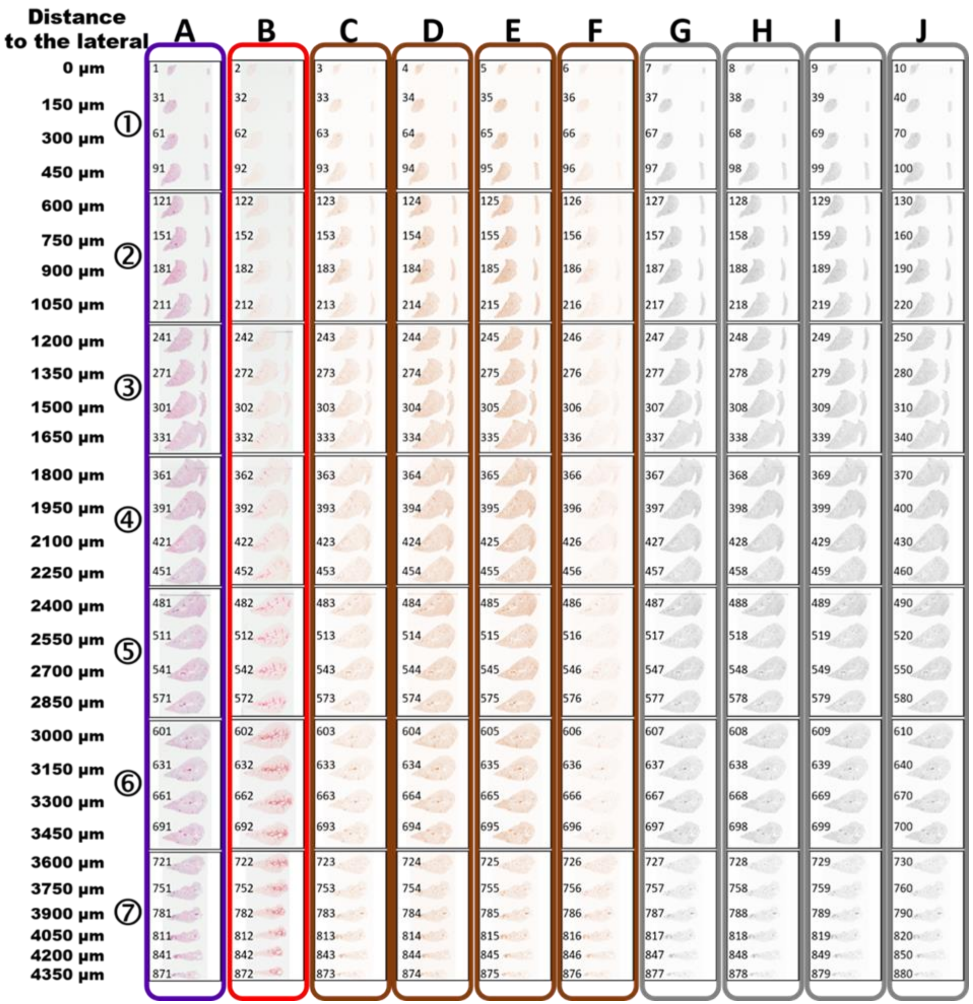

Supplemental Figure 2. Lung tissue sectioning methodology for morphology/pathology analysis in PF rat model

To objectively analyze the pathology of every region in the left lung, the left lungs were serially sectioned into 5-μm slices. For example, in the Normal group, lung sagittal slices numbered from 1 to 10 were placed on slides lettered from A to J, and slices numbered from 11 to 30 were discarded (a total of 20). As the sectioning process was performed toward the hilum, slices numbered from 31 to 40 were placed on slides lettered from A to J, and another 20 slices were discarded. The procedure was repeated until the entire lung was completely sectioned. Thus, slices in column A (numbers: 1, 31, 61, 91, 121,… 871) were all subjected to hematoxylin and eosin (H&E) staining (A); lung slices in column B (numbers: 2, 32, 62, 92, 122,… 872) were stained with Sirius red for the evaluation of tissue fibrosis (B); lung slices in column C (numbers: 3, 33, 63, 93, 123,… 873) were subjected to IHC with anti-ED1 antibody to examine inflammatory responses (C); lung slices in column D (numbers: 4, 34, 64, 94, 124,… 874) were immunostained with anti-proSPC antibody for the labeling of AEC2s (D); lung slices in column E (numbers: 5, 35, 65, 95, 125,… 875) were subjected to immunostaining with anti-α-SMA antibody for the labeling of myofibroblasts (E); and lung slices in column F (numbers: 6, 36, 66, 96, 126,… 876) were subjected to immunostaining with anti-human specific nuclear antibody for labeling of HUMSCs (F). Rows A①–J①, A②–J②, and A③–J③ represent the outermost region of each lobe in a left lung and rows A⑥–J⑥ and A ⑦–J⑦ represent the region close to a hilum of a left lung. The number of lung slices obtained varied between groups (330- 880 slices). The remaining columns (G- J) were preserved as spares.

Supplemental Figure 3

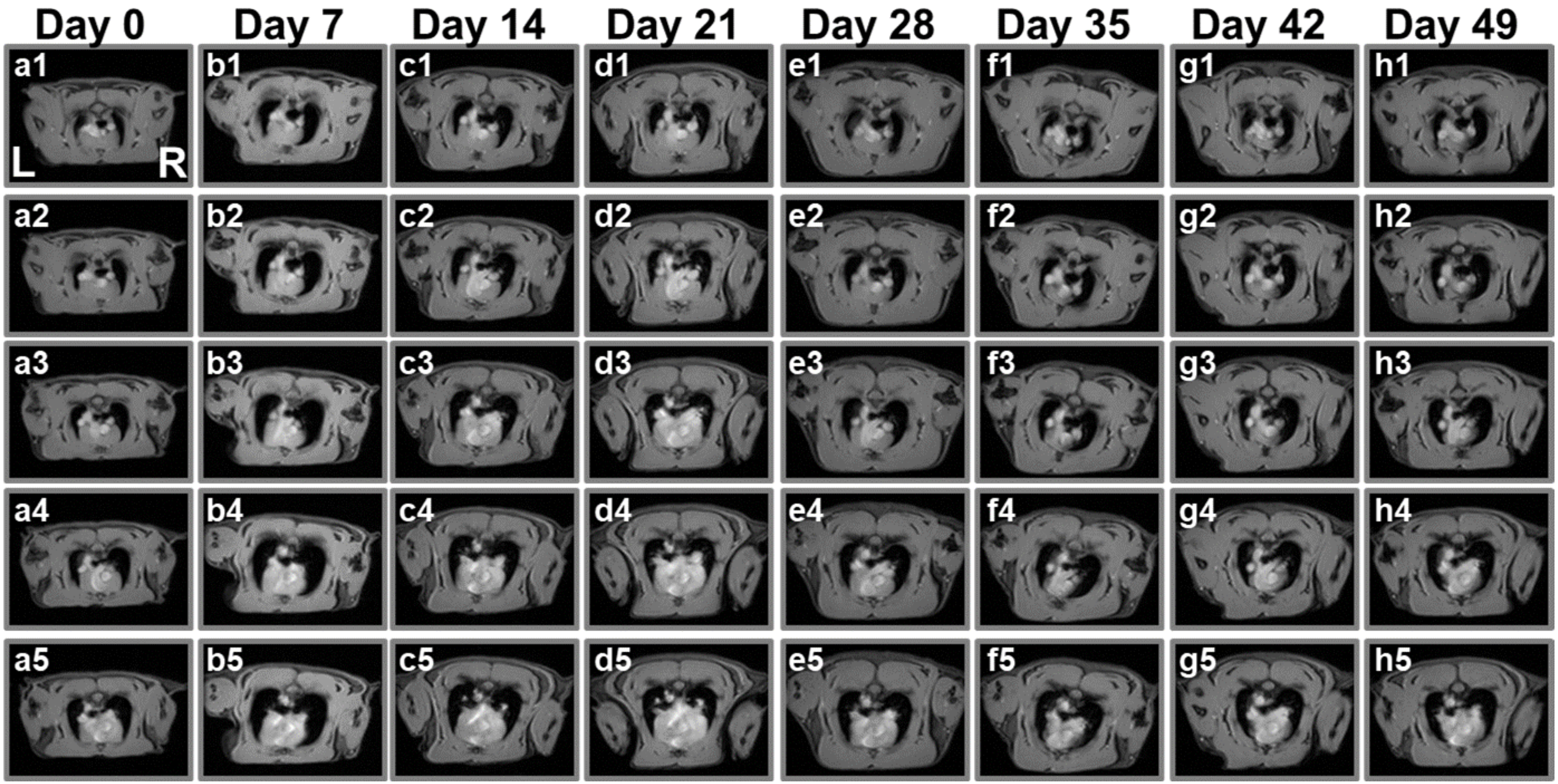

Supplemental Figure 3. MRI scans of rats' thoracic cavities in the Normal group.

Five MRI scans of rats' thoracic cavities in the Normal group. Using carina of the trachea as a landmark for image positioning, five images were used for quantification. Black signals in the thoracic cavity represent space occupied by alveoli. L indicates the left side of the body and R indicates the right. a1–a5: Day 0; b1–b5: Day 7; c1–c5: Day 14; d1–d5: Day 21; e1–e5: Day 28; f1–f5: Day 35; g1–g5: Day 42; h1–h5: Day 49. The space occupied by alveoli is clearly seen in both the left and right lungs in the Normal group.

Supplemental Figure 4

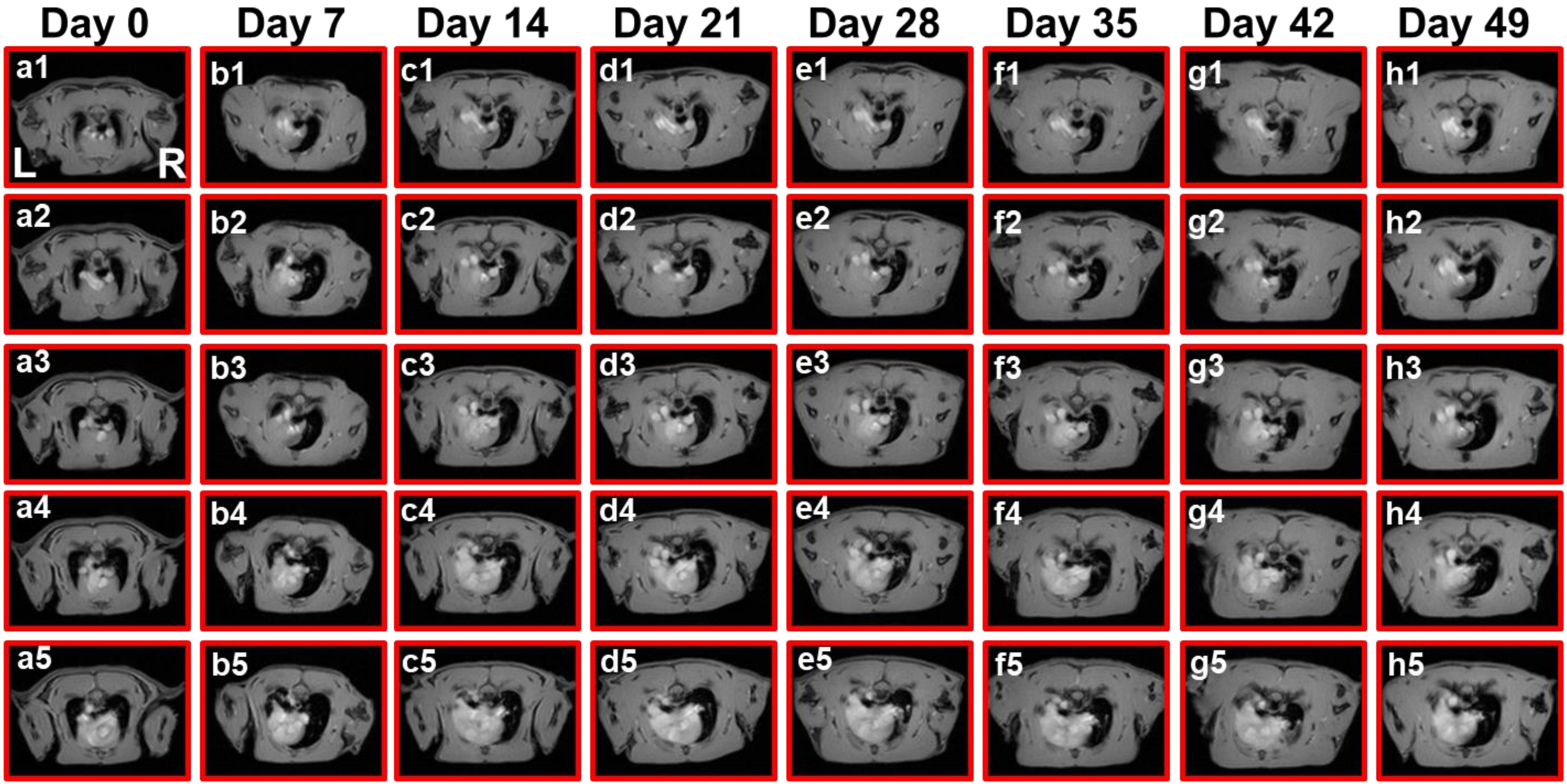

Supplemental Figure 4. MRI scans of the rats' thoracic cavities in the BLM group.

Five MRI scans of the rats' thoracic cavities in the BLM-treated group. On Day 7, alveolar space was significantly reduced in the left lung and white consolidated tissues appeared. On Day 14, the alveolar volume in the left lungs was almost completely lost and had become occupied by consolidated tissue, which was sustained until Day 49.

Supplemental Figure 5

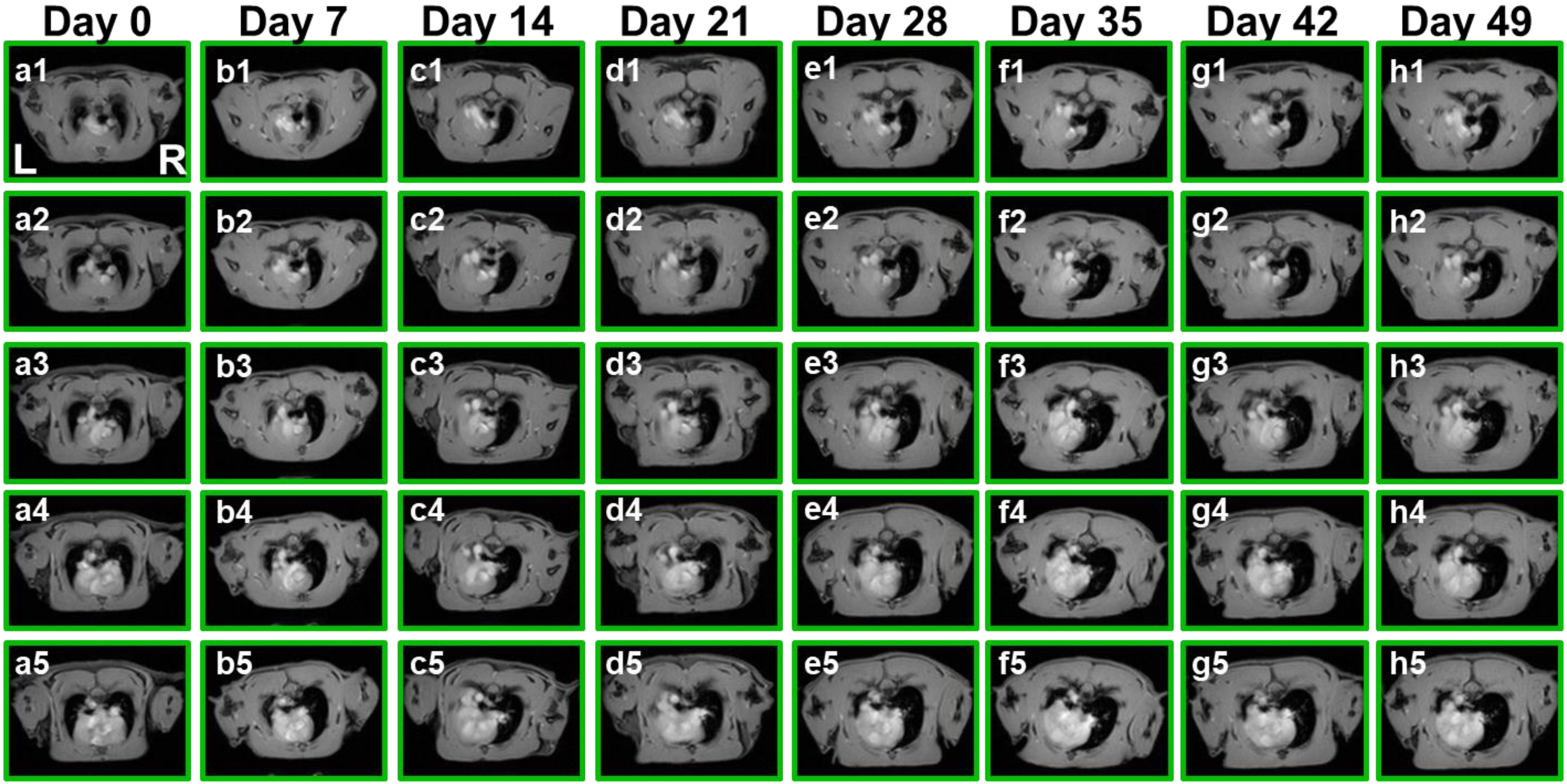

Supplemental Figure 5. MRI scans of the rats' thoracic cavities in the BLM+HUMSCs(LD) group.

Five MRI scans of the thoracic cavities in the BLM+HUMSCs(LD) group are presented.

Supplemental Figure 6

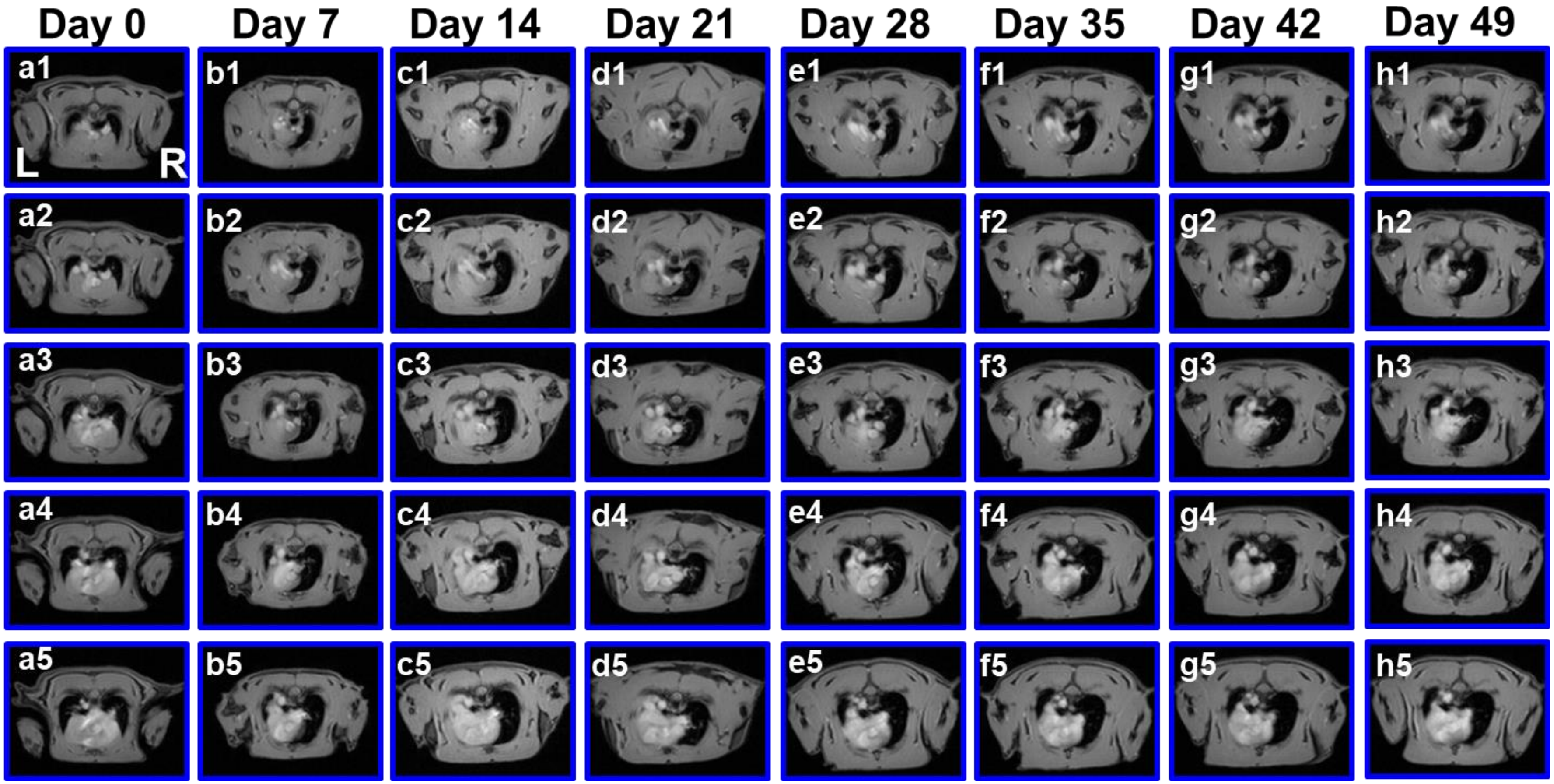

Supplemental Figure 6. MRI scans of the rats' thoracic cavities in the BLM+HUMSCs(HD) group.

Five MRI scans of the thoracic cavities in the BLM+HUMSCs(HD) group are presented.

Supplemental Figure 7

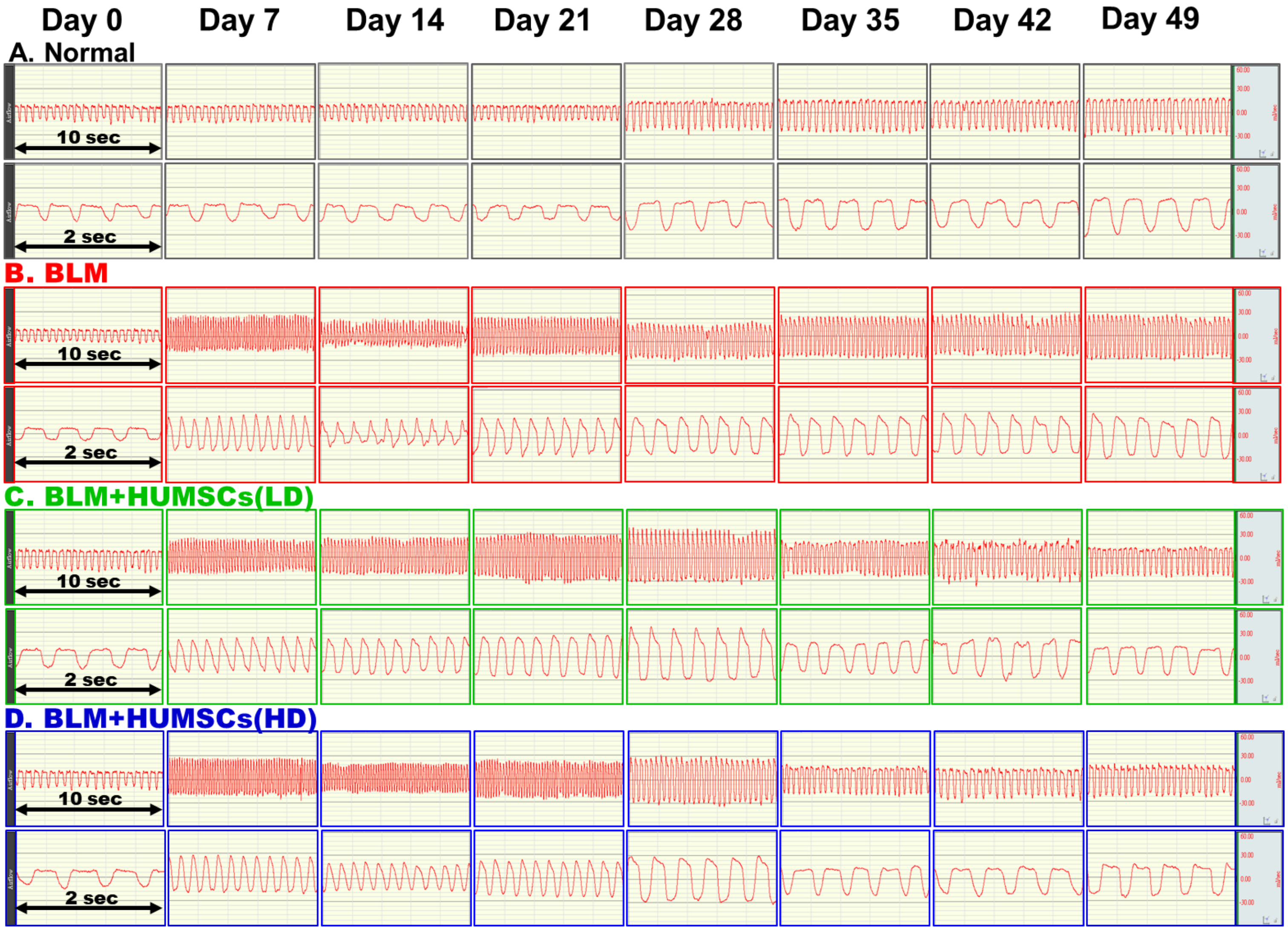

Supplemental Figure 7. Transplantation of HUMSCs alleviated respiratory rates in rats with pulmonary fibrosis.

Respiratory rates were recorded weekly for each group. The respiratory frequency was captured within 10 seconds (upper row) or 2 seconds (lower row) from Day 0 to 49 in the Normal group (A), BLM-treated group (B), BLM+HUMSCs(LD) group (C) and BLM+HUMSCs(HD) group (D).

Supplemental Figure 8

8A. arterial blood oxygen saturation (SpO_2)

	Day 0	Day 7	Day 14	Day 21	Day 28	Day 35	Day 42	Day 49
**Normal (n=17)**	97.2±0.8	97.3±1.2	97.0±0.8	97.2±0.9	97.3±1.3	97.1±1.1	97.4±1.0	97.9±0.6
**BLM (n=25)**	97.2±1.1	82.9±3.2 a	82.1±2.8 a	80.2±3.5 a,b	81.0±2.6 a,b	80.9±2.6 a,b	81.0±2.4 a,b	80.8±2.7 a,b
**BLM +HUMSCs(LD) (n=12)**	97.3±0.9	82.3±2.7 a	82.0±2.2 a	80.4±2.1 a	81.7±2.3 a	83.3±2.7 a,d	84.2±3.2 a,c,d,e	85.3±3.0 a,b,c,d,e
**BLM + HUMSCs(HD) (n=20)**	97.2±0.8	82.8±2.1 a	81.5±1.8 a	80.3±2.8 a,b	84.2±1.7 a,b,c,d	87.6±2.7 a,b,c,d,e	89.9±2.1 a,b,c,d,e,f	92.3±2.3 a,b,c,d,e,f,g

8B. Pulmonary respiratory rate (Breaths per minute; BPM)

	Day 0	Day 7	Day 14	Day 21	Day 28	Day 35	Day 42	Day 49
**Normal (n=17)**	143.5±24.3	137.7±23.3	137.3±18.9	137.2±16.9	140.5±20.3	137.8±24.0	140.7±23.0	139.2±26.4
**BLM (n=25)**	150.2±24.9	303.9±48.7 a	245.6±52.8 a,b	226.3±57.2 a,b	224.1±66.2 a,b	217.0±58.7 a,b,c	207.5±61.8 a,b,c	203.4±54.3 a,b,c
**BLM +HUMSCs(LD) (n=12)**	142.6±27.7	289.3±49.3 a	242.2±47.2 a,b	229.7±32.2 a,b	222.9±64.6 a,b	197.7±62.6 a,b,c	179.2±64.4 b,c,d,e	172.2±58.3 b,c,d,e
**BLM + HUMSCs(HD) (n=20)**	158.8±20.9	313.5±56.4 a	256.0±64.5 a,b	232.6±60.2 a,b	184.4±33.1 b,c,d	175.9±23.6 b,c,d	168.5±26.8 b,c,d	159.0±26.3 b,c,d

Supplemental Fig 8. The statistical comparison of lung function between different time in the same group

a: *vs* D0, b: *vs* D7, c: *vs* D14, d: *vs* D21, e: *vs* D28, f: *vs* D35, g: *vs* D42 in the same group , $p<0.05$.
